# Artificial Intelligence as a Multifaceted Toolkit for Antibacterial Drug Discovery against Resistance

**DOI:** 10.34133/research.1457

**Published:** 2026-11-10

**Authors:** Jifeng You, Shixiang Xu, Baozhang Luo, Shiwei He, Zhihui Yang, Zheng Liu, Yuanyuan Wang, Minghe Wu, Guiling Zhou, Mingming Zhao, Chao Lu, Jialiang Guo

**Affiliations:** ^1^ The Third Affiliated Hospital, School of Medicine, Foshan University, Foshan, Guangdong, China.; ^2^ Foshan Key Laboratory for Drug Screening, Diagnosis, and Treatment of Bacterial and Biofilm Infections, Foshan, Guangdong, China.; ^3^ School of Life Science and Technology, Huazhong University of Science and Technology, Wuhan, Hubei, China.; ^4^ Unit of Pharmaco-Therapy, -Epidemiology and -Economics (PTEE), Department of Pharmacy, University of Groningen, Groningen, the Netherlands.; ^5^ State Key Laboratory of Bioactive Molecules and Druggability Assessment, Guangdong Basic Research Center of Excellence for Natural Bioactive Molecules and Discovery of Innovative Drugs, College of Pharmacy, Jinan University, Guangzhou, Guangdong, China.

## Abstract

How artificial intelligence (AI) can be rationally applied to antibacterial drug discovery and development to overcome antimicrobial resistance (AMR) remains insufficiently defined, particularly across different resistance mechanisms and translational stages. The global emergence and dissemination of AMR are compromising conventional antibacterial therapies, while slow antibiotic discovery, limited target diversity, and reliance on single-mechanism drug design further increase the risk of inadequate treatment options. With strengths in multi-omics integration, complex pattern recognition, and structure-guided molecular design, AI offers a promising approach to support next-generation antibacterial development. This review focuses on AI-related strategies for antimicrobial development against bacterial resistance and provides a systematic overview of advances in target identification, molecular design, activity prediction, and clinical translation. Rather than proposing a new theoretical framework, the review introduces a pragmatic synthesis tool for evaluating where AI adds value and where important limitations remain, considering application objectives, data properties, validation depth, biological interpretability, and translational feasibility. Major challenges are highlighted, including data heterogeneity, clonal bias, limited external validation, discrepancies between in vitro activity and in vivo therapeutic efficacy, and the absence of regulatory approval for any AI-discovered or AI-designed antibacterial agent. Correspondingly, future directions are delineated, including multimodal data integration, explainable AI methodologies, validation through synthetic biology, and coordinated international regulatory efforts. Overall, this review provides a structured overview of how AI can be integrated with rational antibacterial design while considering both its potential and current limitations for addressing multidrug-resistant pathogens.

## Introduction

According to the Global Burden of Disease (GBD) 2021 study, antimicrobial resistance (AMR) was associated with 4.71 million deaths worldwide in 2021, including 1.14 million deaths directly attributable to AMR, and this burden is projected to increase by 2050 [1]. The 2024 WHO Bacterial Priority Pathogens List further highlights clinically important drug-resistant pathogens, including carbapenem-resistant *Enterobacteriaceae* (CRE), methicillin-resistant *Staphylococcus aureus* (MRSA), and multidrug-resistant *Pseudomonas aeruginosa* [2]. Despite continued efforts to develop new antibacterial agents, discovery remains challenging because of bacterial biological complexity, restricted chemical diversity, limited exploration of chemical space, and the continuous emergence of resistance mechanisms. Conventional target-based and empirical screening approaches have historically focused on a relatively narrow set of essential bacterial processes, such as cell-wall synthesis, DNA replication, nucleic acid metabolism, protein synthesis, and membrane homeostasis, potentially limiting target diversity and contributing to repeated exploration of related chemical scaffolds and cross-resistance [3]. These approaches also require substantial experimental resources and provide limited ability to predict pharmacological properties and in vivo efficacy.

Artificial intelligence (AI) has therefore emerged as an increasingly important strategy for integrating heterogeneous biological, genomic, and chemical information in antibacterial discovery and resistance research. As shown in Fig. 1, a macroscopic roadmap of technological evolution is provided. AI approaches have shown potential in resistance prediction, target identification, molecular design, optimization, and candidate prioritization. By enabling the analysis of high-dimensional and heterogeneous datasets, AI can complement conventional discovery strategies and facilitate the identification of biological and chemical patterns that may be difficult to capture through empirical approaches alone. However, AI predictions primarily reflect patterns present in available datasets and therefore remain highly dependent on data quality, biological representation, and model generalizability. Differences in strain composition, experimental protocols, phenotype annotation, and retrospective data collection can introduce systematic bias, while strain-specific biology, clonal bias, limited external validation, and discrepancies between in vitro activity and in vivo therapeutic efficacy may restrict translational utility. Limited interpretability, insufficient prospective evaluation, and challenges in clinical implementation further emphasize that AI should complement rather than replace experimental and clinical evaluation.

**Fig. 1.**
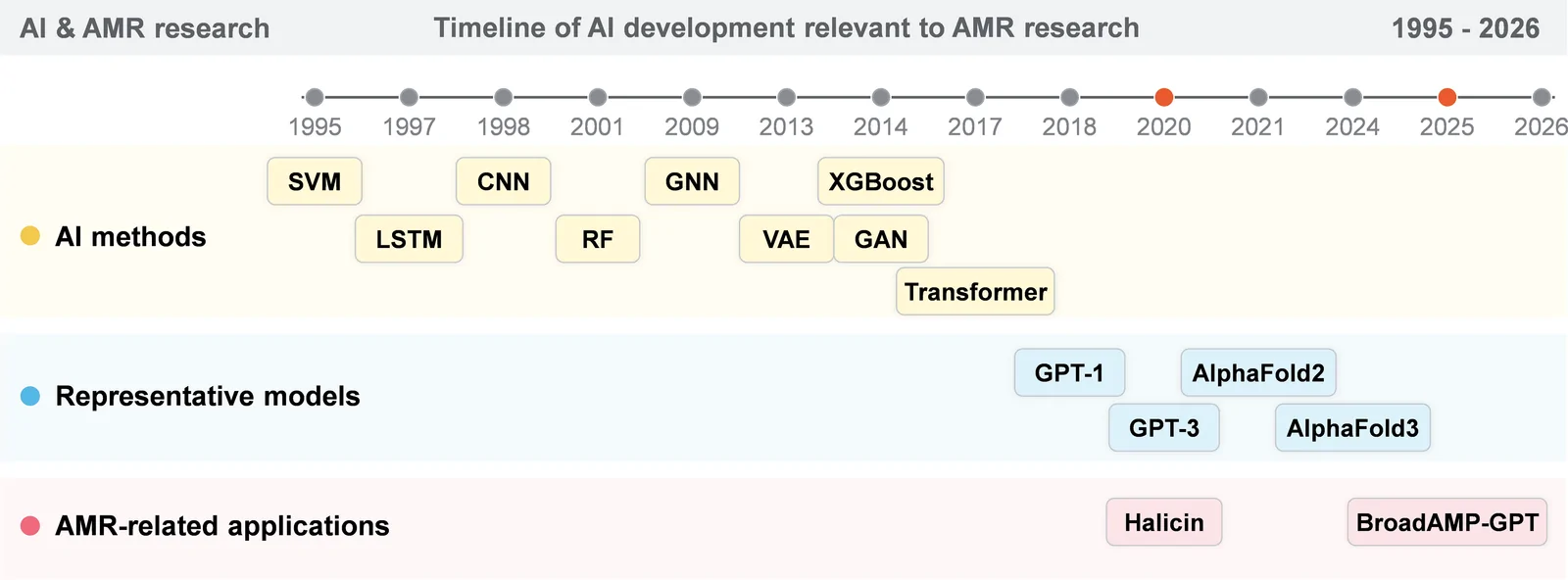
Timeline of the evolution of artificial intelligence paradigms and representative AI-driven applications in AMR research. RF, random forest; CNN, convolutional neural network; LSTM, long short-term memory; XGBoost, extreme gradient boosting; SVM, support vector machine; GNN, graph neural network; VAE, variational autoencoder; GAN, generative adversarial network; GPT, generative pretrained transformer; LLMs, large language models; QSAR modeling, quantitative structure–activity relationship modeling. The indicated years represent the first reports of each method/model.

Previous reviews have established complementary perspectives on AI in antibacterial research. Melo et al. [4] emphasized AI-enabled screening, molecular representation, drug-likeness, and de novo design; Lluka and Stokes [5] focused on how machine learning (ML) can reinvigorate established antibiotic-discovery strategies and support systems biology, combinations, antimicrobial peptides, and mechanism-of-action studies; and Cesaro et al. [6] adopted a broader infectious-disease perspective spanning diagnosis, surveillance, AMR, and therapeutic discovery [4–6]. Wong et al. [7,8] further demonstrated the value of explainable deep learning and benchmarked structure-enabled workflows for antibiotic discovery. Existing works illustrate the diversity of current approaches in terms of research scope, methodological organization, coverage of resistance mechanisms, and consideration of translational challenges (see Table S1 for a broader comparison with representative recent reviews).

The present review uses clinically relevant resistance mechanisms and development stage as a linked organizing axis, separating methodological roles from biological applications and connecting upstream target selection with downstream molecular or regimen design and efficacy prediction. As a narrative review, this article does not aim to provide a systematic quantitative synthesis of the literature; rather, it integrates representative advances, conceptual developments, and translational considerations to offer a comprehensive interpretation of the evolving landscape of AI-driven antibacterial discovery. Rather than proposing a new theoretical framework, it provides a pragmatic assessment tool for evaluating where AI adds value and where important limitations remain. This tool considers application objectives, data properties, validation depth, biological interpretability, and translational feasibility, and distinguishes hypothesis generation from target validation, candidate generation from experimentally confirmed activity, and retrospective prediction from prospective clinical utility. It also incorporates unsuccessful or weakly translating outcomes, including poor cross-dataset generalization, biologically inactive generated candidates, structure-prediction uncertainty, and the persistent in vitro–in vivo–clinical gap. Figure 2 summarizes the application of AI across antibacterial molecular design and related development stages.

**Fig. 2.**
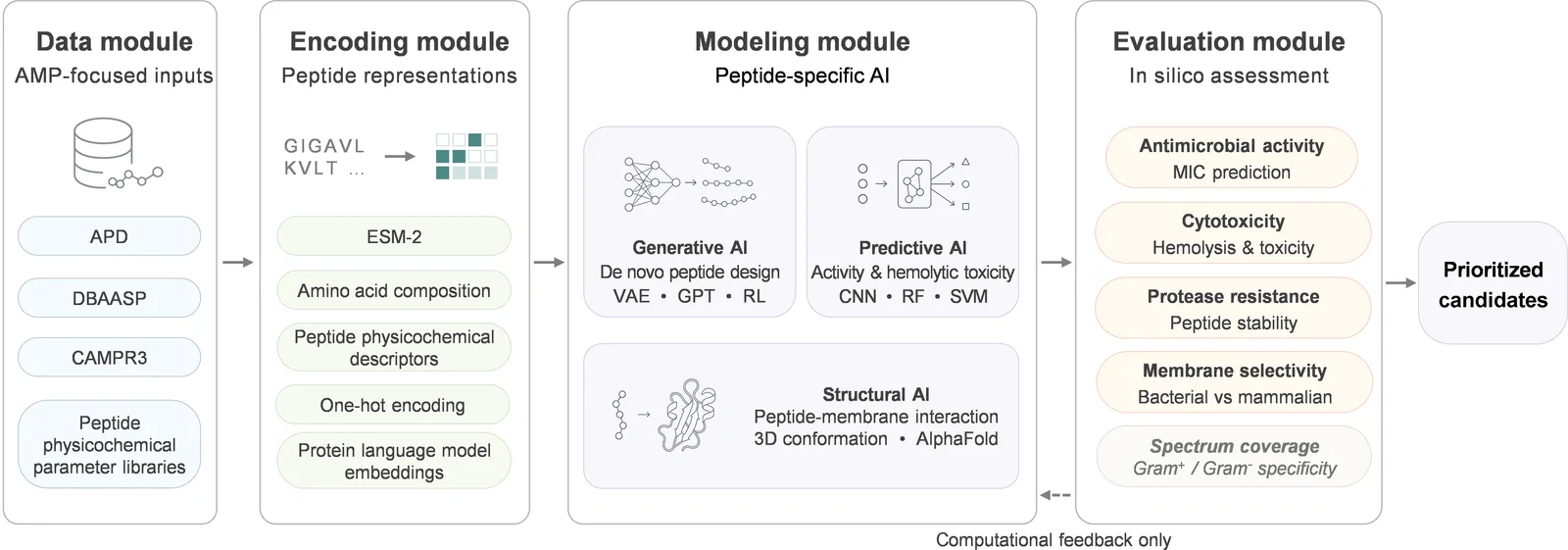
Computational pipeline for AI-assisted antimicrobial peptide discovery and prioritization. AI integrates peptide sequence information, physicochemical properties, and biological activity-related features to enable candidate generation, optimization, and ranking. Prioritized candidates are subsequently advanced for experimental validation and resistance-related characterization.

Accordingly, this review integrates resistance mechanism analysis with AI-assisted target discovery, molecular design, comparative evaluation of AI approaches, and antibacterial efficacy prediction within a unified antibacterial discovery perspective. Figure 3 provides a framework illustrating how AI can support and advance research into resistance mechanisms. The aim is to clarify where AI can provide practical value across the antibacterial development pipeline while identifying the data, biological, experimental, and translational limitations that currently constrain its broader application. AI should therefore be regarded as an integrative decision-support technology that can prioritize hypotheses and candidates, but whose outputs require experimental validation and, ultimately, clinical evaluation.

**Fig. 3.**
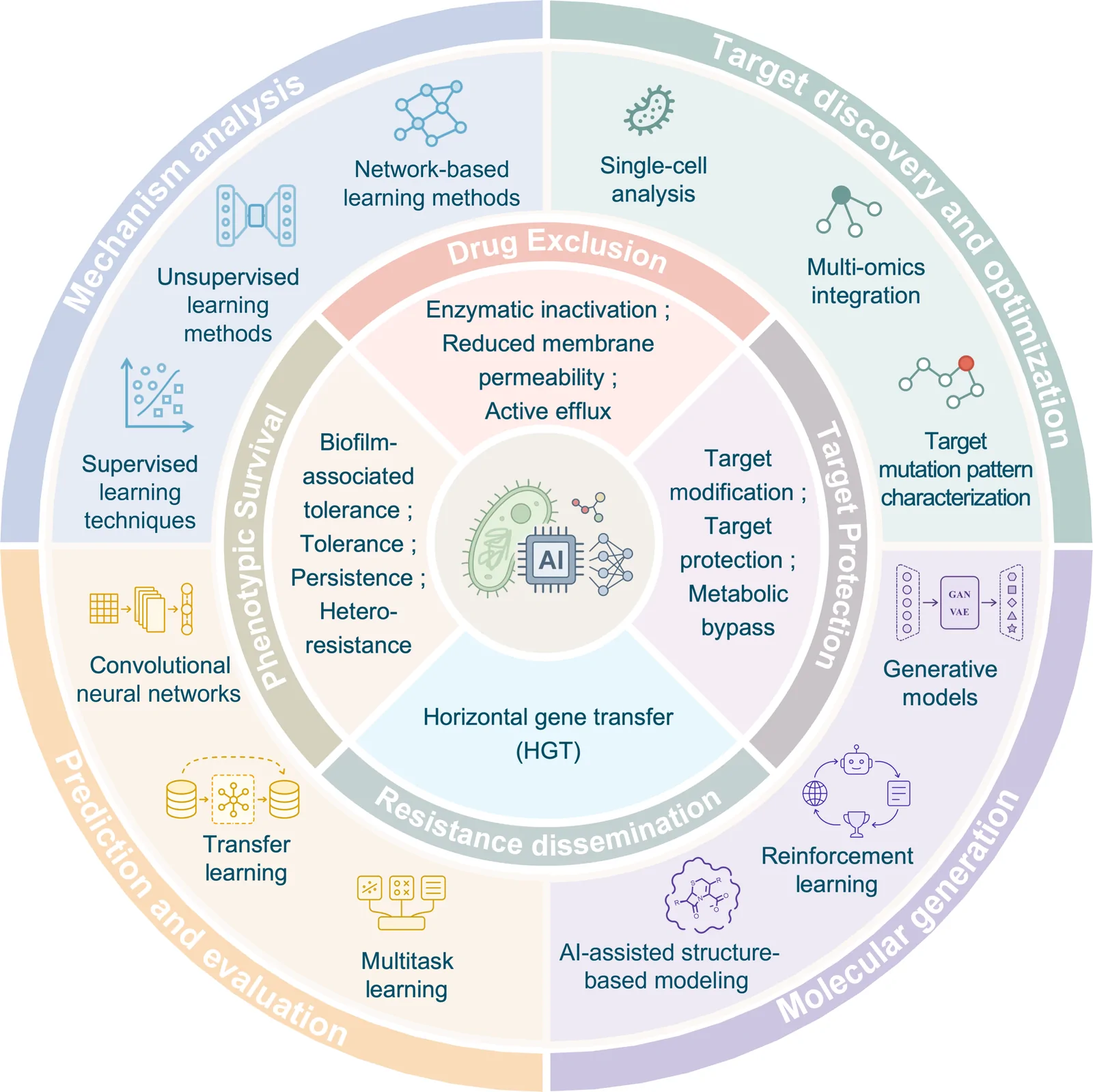
AI-enabled framework for AMR research and antibacterial discovery. AI integrates biological, molecular, and phenotypic information to support resistance mechanism analysis, target identification, molecular generation, and predictive evaluation, providing a unified framework for advancing antibacterial discovery.

## Resistance Mechanisms and Methodological Roles of AI

### Classification of core mechanisms of bacterial resistance

Bacterial resistance develops through dynamic interactions among genetic determinants, antibacterial selective pressures, and bacterial adaptive responses. Beyond well-established mechanisms such as enzymatic inactivation, target modification, efflux, and biofilm-associated tolerance [9,10], resistance can also arise from altered permeability, target protection, metabolic bypass, and horizontal gene transfer (HGT) [11,12]. Collectively, these mechanisms can decrease intracellular drug exposure and maintain bacterial survival by preserving target function, bypassing antibiotic inhibition, or promoting the acquisition and spread of resistance determinants.

Beyond genetic resistance mechanisms, phenotypic survival strategies also contribute to treatment failure. Tolerance enables transient survival during antibiotic exposure, whereas persistence involves dormant subpopulations that resume growth after antibiotic exposure is discontinued and may contribute to recurrent infections [13,14]. Heteroresistance refers to bacterial populations containing a minority fraction with elevated resistance phenotypes that may escape routine susceptibility testing [15]. These mechanisms frequently coexist within clinical isolates and may limit the effectiveness of antibacterial strategies that rely on a single target [16,17].

### Methodological classification and roles of AI in antibacterial resistance research

To distinguish AI from the broader field of computational biology, this review uses AI to denote learning or generative models that infer representations, decision rules, or molecular distributions from data. These include classical ML methods such as random forests, XGBoost, and support vector machines; deep-learning architectures such as convolutional neural networks (CNNs) and graph neural networks (GNNs); and generative or foundation models including variational autoencoders (VAEs), generative adversarial networks (GANs), diffusion models, reinforcement learning (RL), transformers, and protein-language models. Uniform manifold approximation and projection (UMAP) and weighted gene co-expression network analysis (WGCNA) are treated as complementary analytical methods, whereas conventional molecular docking, molecular dynamics, and pharmacokinetic/pharmacodynamic (PK/PD) simulations are non-AI computational tools. AlphaFold2 is an AI structure predictor, but subsequent docking of its predicted structures is not itself AI-driven. Methodologically, AI contributes mainly to mechanism inference, candidate-space exploration, and resistance or treatment prediction.

#### Learning-based approaches for resistance mechanism inference

AI-based mechanism inference integrates genomic, transcriptomic, proteomic, phenotypic, and clinical data to prioritize resistance-associated features and connect molecular variation with phenotype. Classical models remain effective for structured datasets with predefined variables, whereas deep or network-based approaches can be advantageous for higher-dimensional nonlinear relationships [18,19]. Their main output is a testable mechanistic hypothesis, not proof of causality; feature importance or predictive association must therefore be followed by functional validation.

#### Generative and optimization approaches for candidate-space exploration

Conventional antimicrobial peptide (AMP) discovery combines experimental screening, database mining, and virtual screening, but efficient identification of active candidates from large peptide spaces remains challenging [20]. Generative and optimization models explore chemical or peptide space under activity, structural, and developability constraints [21,22]. GNNs and end-to-end representation learning are most likely to outperform descriptor-based quantitative structure–activity relationship (QSAR) or random forests when training sets are sufficiently large and diverse and molecular topology or interaction context carries information not captured by predefined descriptors; for smaller, well-characterized datasets, classical models may be equally or more robust [23,24]. Likewise, generative workflows are most useful when candidate generation is coupled to predictive filtering and experimental validation, as illustrated by integrated discovery pipelines such as NG1 (shown in Fig. 4) [25].

**Fig. 4.**
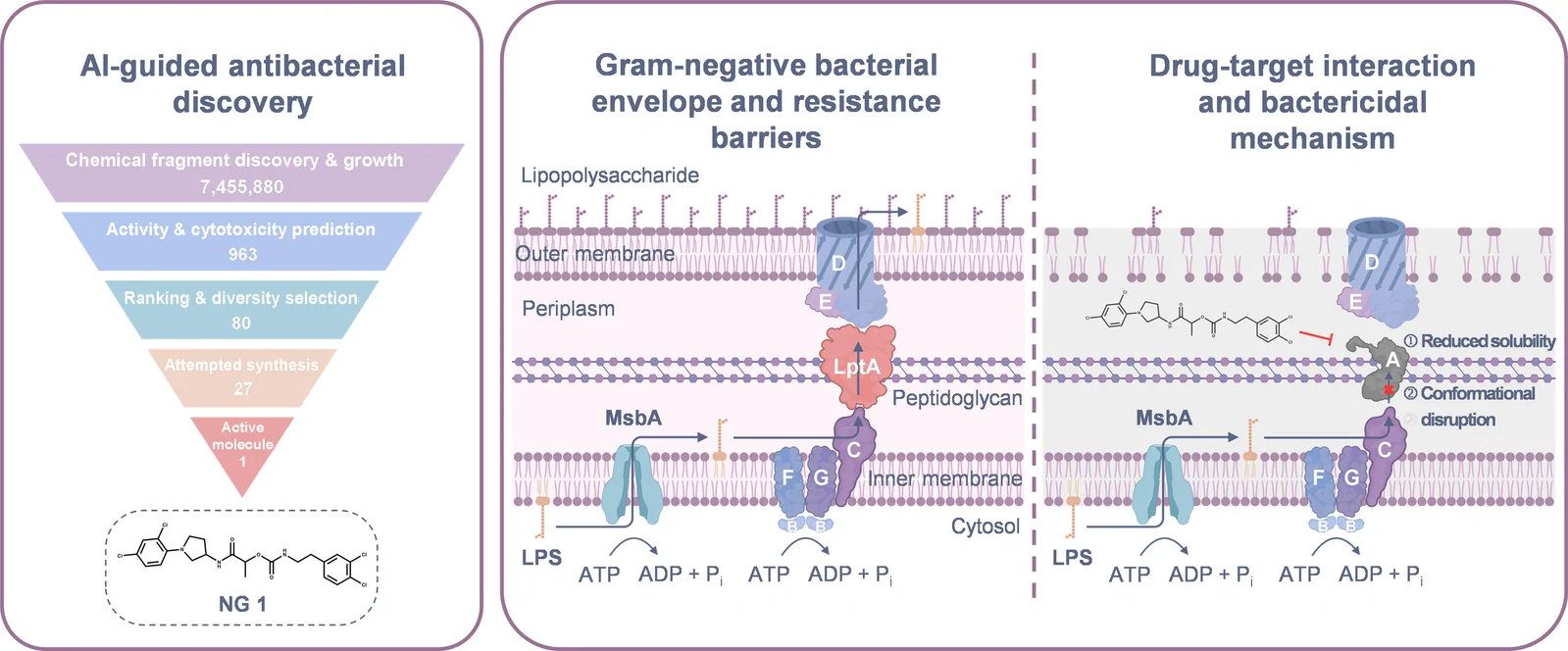
AI-guided de novo discovery of NG1 and mechanistic investigation of its antibacterial activity. NG1 was identified through graph neural network-based screening and generative molecular design as a de novo antibacterial candidate against multidrug-resistant *Neisseria gonorrhoeae*. Mechanistic studies suggested that NG1 targets LptA, disrupts lipooligosaccharide transport, and impairs outer-membrane integrity. In the schematic representation of the Lpt transport pathway, D, E, C, F, G, and B denote LptD, LptE, LptC, LptF, LptG, and LptB respectively.

#### Predictive models for resistance phenotypes and treatment response

Predictive models differ from mechanism-inference and molecular-generation approaches because their primary output is a probability, class, or quantitative estimate for a defined biological or clinical endpoint. CNNs can extract resistance-related features from matrix-assisted laser desorption/ionization mass spectrometry (MALDI-MS) spectra. Genome-based predictors such as GenTB and resistance-gene annotation resources such as hierarchical multi-task deep learning framework for antibiotic resistance gene annotation (HMD-ARG) illustrate complementary endpoints, namely, phenotype-oriented decision support versus mechanistic annotation, while sequence-based and longitudinal models support genomic resistance prediction, surveillance, and evolutionary forecasting [26–29]. Performance should therefore be judged against the intended endpoint and the population in which the model must generalize, rather than by algorithm family alone.

#### Emerging AI paradigms: Explainable AI, foundation models, large language models, and agentic AI

Emerging paradigms mainly add enabling capabilities to established workflows. Explainable AI can identify features contributing to predictions, protein-language and foundation models can transfer sequence or molecular representations to data-limited tasks, large language models (LLMs) can support peptide generation or structured knowledge extraction, and agentic systems aim to connect hypothesis generation, prediction, and experimental planning [7,18,19,30]. These emerging paradigms already have concrete applications in AMP design. BroadAMP-GPT uses a generative pretrained transformer (GPT)-based language model to generate broad-spectrum AMPs with activity against multidrug-resistant ESKAPE (*Enterococcus faecium*, *Staphylococcus aureus*, *Klebsiella pneumoniae*, *Acinetobacter baumannii*, *Pseudomonas aeruginosa*, and *Enterobacter* spp.) pathogens [31], while a latent diffusion model has been applied to expand peptide sequence space and generate diverse, potent AMP candidates [32]. These benefits also introduce distinct limitations, as explanations are not necessarily causal, foundation models may inherit biases from their pretraining data, generative systems may produce plausible yet inactive candidates, and agentic workflows can raise concerns regarding reproducibility, validation, and safety. As summarized in Table 1, no method is optimal across all stages, and method complexity is justified only when it adds measurable value over simpler baselines.

**Table 1.** Simplified comparison of core AI technologies for multidrug-resistance (MDR) antibacterial research

AI approach/modeling strategy	Example models	Main MDR-related applications	Core advantages	Key limitations	Translational readiness	Data requirement	Validation level
Classical machine learning	RF, XGBoost, SVM	Genomic resistance prediction, strain typing	High interpretability, small-data fit	Cannot simulate 3D protein interactions	Relatively mature	Labeled compounds or genomic profiles (500–5,000); tabular descriptors [192]	Cross-validation and external test set; in vitro MIC; limited in vivo [161]
Convolutional neural networks	CNN	MALDI-MS resistance screening, biofilm analysis	Fast high-throughput detection	Poor cross-laboratory generalization	Relatively mature	Thousands of labeled mass spectra or images; standardized preprocessing [29]	Independent cohort validation; in vitro concordance; limited multi-center [139]
Graph neural networks	DTBind	Mutated target-binding prediction	Captures pocket structural changes	High computing cost	Emerging	Protein-ligand binding pairs (BindingDB or PDBbind); 3D structures; GPU-intensive [21]	Scaffold-based splitting; in vitro IC_50_ or *K*_i_; limited prospective screening [193]
Generative AI	VAE, GAN, PepGAN	De novo AMP design	Diverse novel molecular skeletons	Mode collapse, high hemotoxicity	Early stage	Thousands of AMP sequences from curated databases (DBAASP or APD3); small imbalanced data [34]	In silico then in vitro MIC and hemotoxicity; selected in vivo; high toxicity attrition [22]
Reinforcement learning	AMPainter	Multi-objective lead optimization	Custom therapeutic optimization	Sparse reward, strain overfitting	Moderate	5,000 or more compounds with full bioactivity data; reward function design [108]	In silico optimization then in vitro activity and selectivity; limited in vivo [35]
Protein language and sequence models	ESM-2, BroadAMP-GPT, LSTM	Resistance-gene annotation, peptide prediction	Zero/few-shot generalization	Domain data shift risk	Relatively mature	Millions of protein sequences (UniProt or MGnify) for pretraining; hundreds to thousands of AMP labels for fine-tuning [35]	Zero-shot or few-shot benchmark; in vitro MIC; limited prospective validation [31]
Equivariant diffusion	DiffLinker	3D pocket adaptive molecule design	High-fidelity 3D generation	Slow sampling, heavy GPU load	Early stage	3D protein pocket structures; large-scale conformer datasets; SE(3)-equivariant GPU [104]	In silico 3D generation and docking; limited in vitro binding [118]
Multi-task learning	M3-CAD, APEX	Multi-mechanism AMP design	Alleviates small-data overfitting	Task conflict reduces accuracy	Emerging	Multi-task labeled datasets sharing representations; hundreds to thousands per task [113]	Cross-task validation and in vitro MIC; limited in vivo [27]

These model classes are suited to different stages of antibacterial discovery. GNNs are most useful when molecular topology or interaction relationships are central to prediction, particularly for drug-target recognition or graph-structured molecular data [21,24]. VAEs provide a continuous latent representation that facilitates controllable exploration and optimization of molecular space [33], whereas GANs are more suitable for activity-guided generation and expansion of candidate diversity [34]. RL is particularly useful for multi-objective optimization when antibacterial activity, selectivity, synthetic accessibility, and other properties must be balanced simultaneously [23]. Transformers and protein-language or foundation models are better suited to large-scale sequence representation and transfer to downstream tasks, particularly when task-specific labeled data are limited [31,35]. Thus, model selection should be determined by data structure, development objective, and validation requirements rather than model complexity alone.

### Selection of AI strategies across antibacterial development stages

AI strategy selection should follow the maturity of biological evidence and the intended decision. Poorly characterized resistance states favor multi-omics or single-cell approaches for hypothesis generation; established genotype–phenotype relationships support supervised prediction and target prioritization, well-characterized targets can justify structure-informed optimization, and emerging population-level resistance patterns are better addressed by surveillance or forecasting models [36–38]. Thus, AI strategies should be selected according to the maturity of biological evidence and the specific research objective.

The integration of AI with emerging biological technologies, including single-cell sequencing and multi-omics analysis, may further expand the identification of previously unrecognized resistance mechanisms. However, their performance remains sensitive to differences in bacterial species, strain backgrounds, experimental procedures, data quality, and biological complexity, which can limit model reliability and generalizability. More importantly, computational success does not necessarily translate into therapeutic success. AI-guided candidates with promising in vitro activity may subsequently show poor PK properties, host toxicity, limited in vivo efficacy, or rapid resistance development [39]. Generative models may also produce chemically novel candidates with favorable computational scores that are difficult to synthesize or prove microbiologically inactive during experimental validation [40].

Similar limitations apply to predictive and structure-based approaches. Genomic resistance models trained on limited strain collections may show reduced performance in geographically or phylogenetically distinct populations, highlighting the importance of dataset diversity and external validation [41]. Although protein structure prediction has expanded access to structural information, predicted structures do not necessarily ensure accurate ligand-binding assessment, and docking-based applications may still require complementary rescoring and experimental confirmation [42]. Overall, current AI approaches are more appropriately regarded as tools for hypothesis generation, candidate prioritization, and iterative optimization within integrated computational and experimental workflows rather than as independently validated platforms that can replace microbiological and pharmacological evaluation [43].

## Application and Evaluation of AI in Antibacterial Drug Target Discovery

Accurate identification and optimization of antibacterial targets are essential for addressing drug resistance. Compared with conventional experimental approaches, AI can integrate genomic, structural, and pharmacological data to prioritize targets and predict target–compound interactions [44]. As shown in Fig. 5, major applications include protein–drug interaction analysis, protein structure prediction, synthetic protein design, and functional prediction [45,46]. Structural tools such as AlphaFold2 can also provide useful information when experimental structures are unavailable, particularly when combined with complementary computational and experimental validation [8]. Accordingly, AI-assisted antibacterial target discovery can be broadly organized into 3 complementary strategies, including optimization of established resistance-associated targets, discovery of novel resistance-associated targets, and identification of targets for restoring antibacterial susceptibility.

**Fig. 5.**
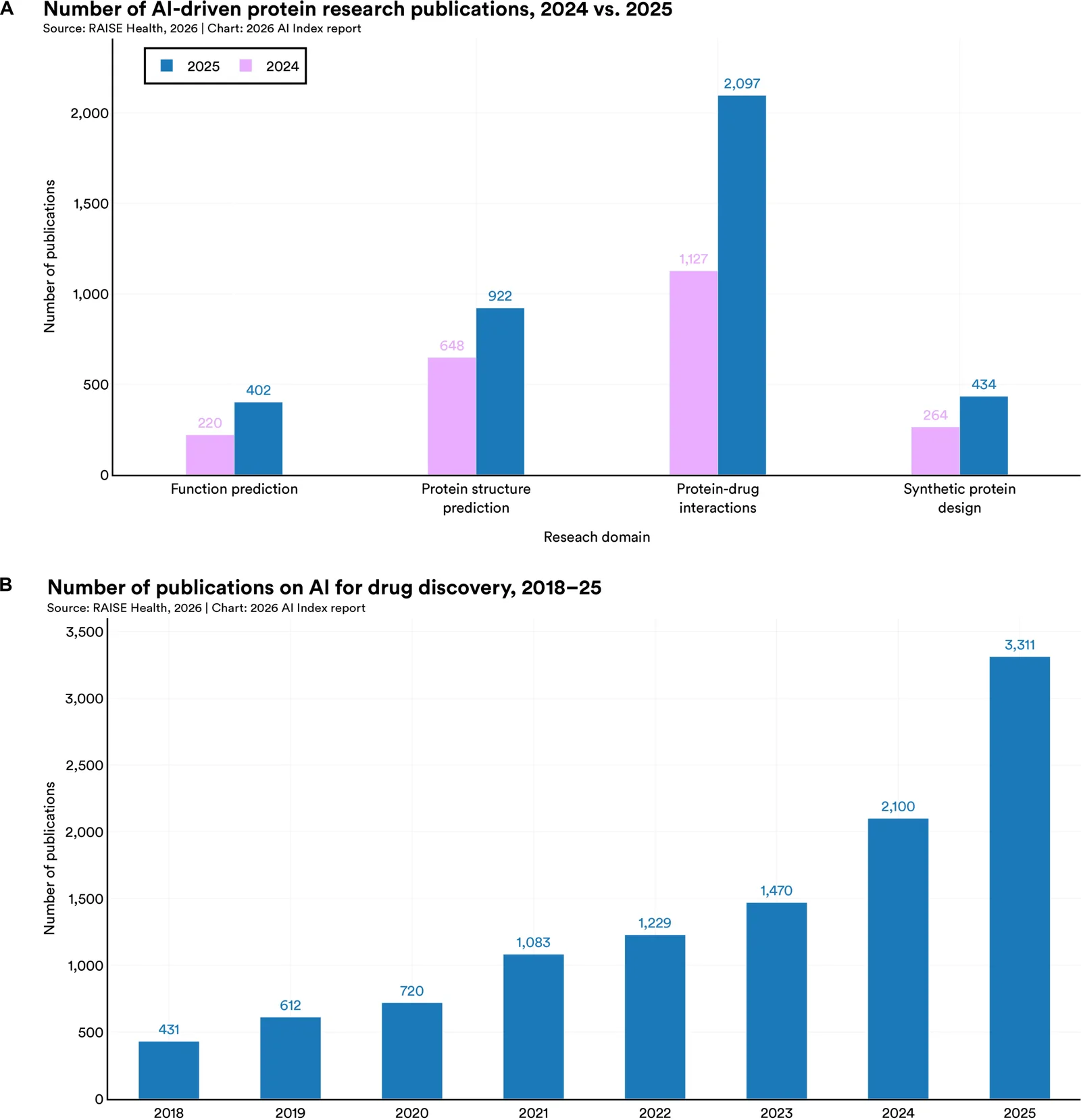
Publication trends of AI-driven drug discovery and protein research. (A) AI-driven publications in 4 protein research domains in 2024 and 2025, including function prediction, protein structure prediction, protein–drug interactions, and synthetic protein design. (B) Annual publication volume for AI in drug discovery from 2018 to 2025. Reproduced with permission from Ref. [46] under the Creative Commons Attribution-NoDerivatives 4.0 International License (CC BY-ND 4.0).

### AI-guided optimization of traditional drug resistance targets

Well-characterized targets such as PBP2a and β-lactamases provide a relatively mature setting for AI-guided optimization because resistance mechanisms, sequence variation, and structural information are often available [47]. Mutation prioritization and target-binding analysis are best viewed as consecutive steps within the same optimization workflow rather than as separate discovery pathways.

#### AI-driven characterization of target mutation patterns

Supervised learning methods, including random forest and XGBoost, integrate bacterial genomic or mutation features with antibacterial susceptibility data to identify variants associated with altered drug responses, whereas sequence-based models such as hidden Markov models are better suited for detecting conserved and variable residues within resistance-related protein families [48,49]. These approaches bridge mutation prioritization with structural and functional interpretation. TB-AMRpred represents a phenotype-associated application that identifies known and candidate resistance mutations from paired genomic and susceptibility data in *Mycobacterium tuberculosis* [50]. In *Escherichia coli*, the integration of ML with genome-scale metabolic models extends mutation analysis beyond individual variants toward network-level resistance determinants [51], while related predictive strategies may facilitate downstream antibacterial sequence optimization after informative resistance-associated features have been identified [52]. However, the reliability of AI-based mutation analysis depends strongly on the accurate representation of genotype–phenotype relationships across diverse bacterial lineages and resistance backgrounds [53,54]. Therefore, these approaches are currently most valuable for prioritizing experimentally testable variants and generating mechanistic hypotheses rather than independently establishing causal resistance mechanisms.

#### AI-assisted target-binding analysis and redesign

Resistance-associated mutations can reshape binding-site geometry and weaken target–drug interactions, but the computational roles within this workflow should be separated. Conventional docking and molecular dynamics are structure- and physics-based tools for comparing ligand compatibility across alternative conformations, whereas ML rescoring or learned interaction models constitute the AI component [55,56]. Studies spanning omics-informed target prioritization and mutation-specific virtual screening collectively indicate that these approaches are most useful for narrowing candidates and generating testable structural hypotheses rather than establishing antibacterial activity on their own [57,58]. Deep-learning structure predictors such as AlphaFold2 can expand structural coverage [59], but predicted structures do not guarantee accurate ligand-binding assessment, and AlphaFold2-based docking can remain unreliable even when ML rescoring improves rankings [8]. Thus, the value of an AI-assisted structural workflow depends on the quality of the structural model, compound-space coverage, and experimental confirmation [60,61].

### AI-driven discovery of novel resistance-associated targets

Novel target discovery is most useful when resistance biology is incompletely characterized and candidate vulnerabilities are not obvious [62]. Multi-omics integration and single-cell analysis provide complementary perspectives, with multi-omics approaches capturing coordinated network-level states and single-cell analysis resolving rare or transient subpopulations that may be overlooked in bulk measurements.

#### Target discovery based on multi-omics integration

Learning-based multi-omics methods can integrate genomic, transcriptional, metabolic, and phenotypic information, while WGCNA and related correlation-network methods serve as complementary non-AI analyses [63]. Compared with single-gene analysis or conventional genome-wide association approaches, the recurring value across *E. coli* and cross-species studies is the shift from isolated determinants toward network-level resistance modules and coordinated biological states [64,65]. This broader view can improve target prioritization, but its reliability depends on compatibility among datasets, annotation quality, and subsequent functional validation because network association does not by itself establish causality.

#### Rare target discovery based on single-cell sequencing

AI-assisted bacterial single-cell analysis enables the detection of rare resistant or drug-tolerant subpopulations that may remain unresolved in bulk sequencing approaches. Bacterial single-cell RNA sequencing (scRNA-seq) is not itself an AI technology but provides high-dimensional data that can be analyzed using ML and other computational approaches to detect rare resistant or drug-tolerant states obscured in bulk measurements. Platforms such as PETRI-seq, microSPLiT, BacDrop, and M3-seq have expanded the resolution and throughput of bacterial single-cell profiling [66,67]. Compared with multi-omics integration, single-cell approaches are better suited to resolving cell-to-cell heterogeneity, transient stress responses, and uncommon subpopulations. BacDrop and M3-seq illustrate complementary strengths, respectively resolving antibiotic-associated heterogeneous states and enabling scalable detection of rare bacterial populations [68,69]. These platforms are experimental data-generation tools rather than AI models, and their value for AI-assisted target discovery lies in supplying single-cell datasets for computational prioritization of unusual states or candidate regulators. Integration with genome-wide CRISPRi screening further links rare-state detection to functional validation, as demonstrated for persistence regulators in *E. coli* [70]. However, bacterial scRNA-seq remains limited by low mRNA abundance, incomplete transcript capture, and species-dependent differences in cell-wall structure and sample processing, restricting sensitivity and cross-species applicability. Improved MATQ-seq still captures only part of the transcriptome, while broader use of BacDrop requires further optimization of permeabilization and transcript-capture strategies [68,71,72]. Thus, candidate targets from single-cell analyses should be treated as hypotheses requiring independent functional validation.

### AI-driven discovery of resistance-reversal targets for restoring antibacterial susceptibility

Resistance-reversal target discovery seeks to restore susceptibility by interfering with regulatory or accessory processes that maintain resistance, including efflux control, biofilm physiology, and resistance-gene expression [73,74]. Compared with conventional essential-gene targeting, this strategy is attractive when an existing antibiotic can be potentiated, but it requires evidence that the predicted regulator is functionally actionable rather than merely correlated with resistance.

#### AI-based discovery of efflux pump and biofilm disruption targets

Efflux pump regulation and biofilm-associated resistance involve complex regulatory networks that are difficult to resolve by single-variable analyses. AI-assisted integration of transcriptomic, genomic, proteomic, metabolomic, and phenotypic data can prioritize resistance-related regulators and functional sites. In *P. aeruginosa*, such approaches have identified antibiotic-responsive regulatory modules and functionally relevant MexB residues [75,76]. For biofilm resistance, TargIDe predicts targets from compounds with confirmed antibiofilm activity, while computational screening has identified LasR-targeted inhibitors [20,77,78]. Multimodal profiling can further link biofilm phenotypes with regulatory determinants and susceptibility patterns. These approaches support mechanism-oriented target prioritization, although predicted targets still require genetic or pharmacological validation and evaluation with conventional antibacterials or antibiofilm adjuvants [79].

#### AI-guided discovery of resistance-gene silencing targets

Resistance-gene silencing offers a more sequence-specific reversal strategy. TargetRNA3 and sRNAdeep support prediction of bacterial small RNA (sRNA) interactions and regulatory genes, while computational optimization of crRNA–Cas13 interactions extends the concept to programmable transcript suppression [80–82]. The main bottleneck is no longer target prediction alone but delivery and intracellular function, including stability, off-target activity, variable expression level, and inefficient bacterial entry, which can erase the advantage of a highly specific computational design [83]. These resistance-reversal routes also differ in translational tractability. Efflux or quorum-sensing regulators may be accessible to small-molecule inhibition but can have network-level pleiotropic effects, whereas RNA or CRISPR-based silencing offers sequence specificity at the cost of a more difficult delivery problem. Consequently, computational target confidence should not be separated from intervention feasibility. A regulator with strong omics support may still be a poor therapeutic target if intracellular exposure cannot be achieved, while a moderately ranked but chemically tractable accessory target may offer a more realistic route to restore antibiotic susceptibility.

### Integrated assessment and selection strategy for AI target discovery technologies

Based on the comparative analysis above, Fig. 6 provides a pragmatic synthesis of AI-assisted target discovery strategies. Its role is primarily upstream, namely, to determine which resistance-associated target or regulatory process is sufficiently supported to justify subsequent intervention development. It organizes current approaches into 3 complementary categories according to the maturity of biological evidence [84,85]. Traditional target optimization is most suitable when resistance-associated targets have been established and sufficient structural or mutational information supports further refinement [86]. AMR-reversal target discovery is particularly valuable when existing antibiotics can be potentiated through interference with regulatory or accessory mechanisms, including efflux activity, biofilm formation, and adaptive resistance pathways [87]. By contrast, novel target discovery is mainly applicable to poorly characterized resistance mechanisms or biological contexts in which previously unrecognized therapeutic vulnerabilities remain to be explored [88].

**Fig. 6.**
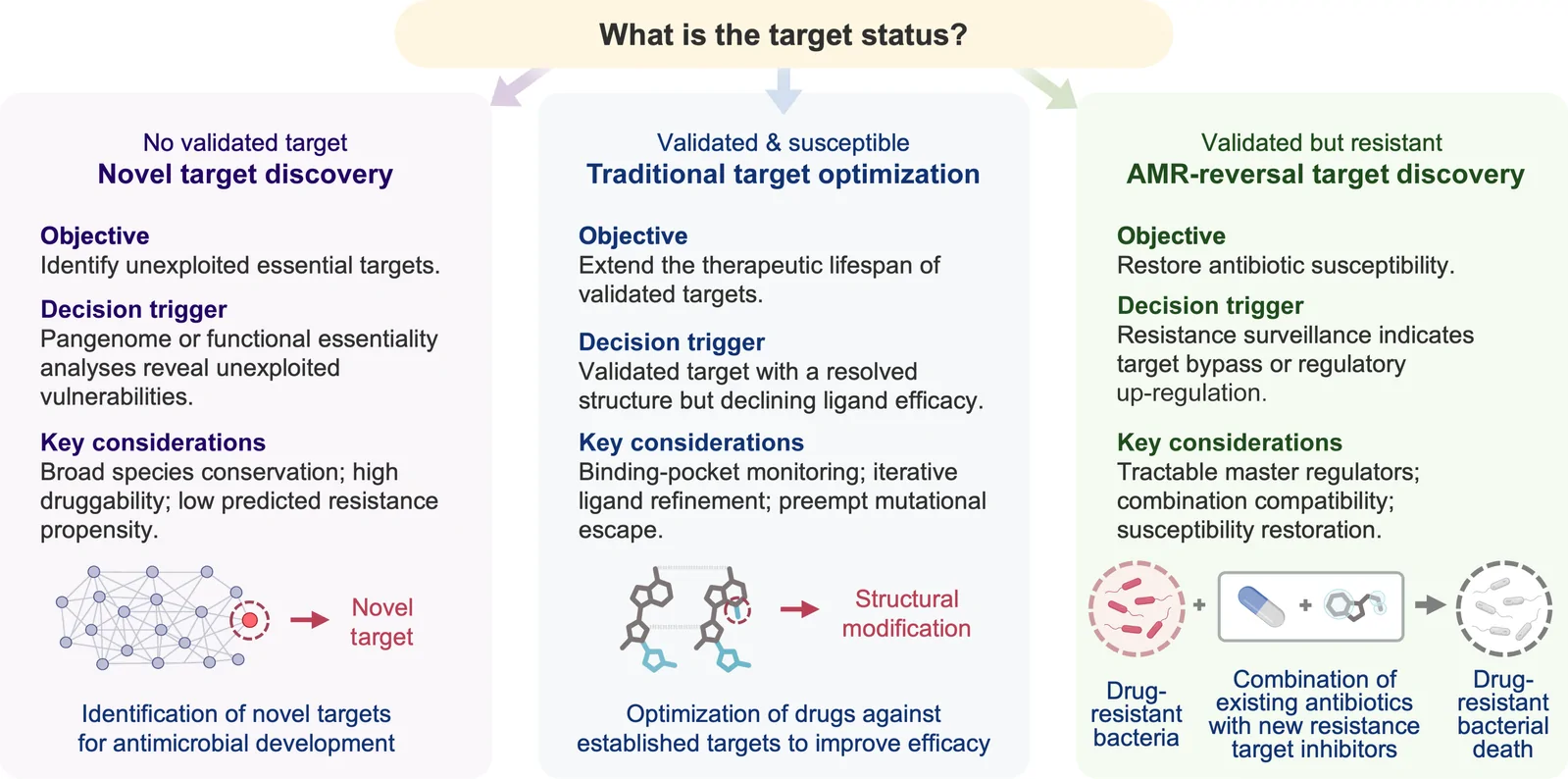
AI-assisted strategies for antibacterial target discovery and optimization in antibacterial resistance research. Three complementary routes are presented, including AMR-reversal target discovery, novel target discovery, and traditional target optimization. AMR-reversal target discovery uses learning-based or network-informed analyses to prioritize regulatory mechanisms that may restore antibiotic susceptibility. Novel target discovery can integrate single-cell or multi-omics data with learning models to identify candidate targets. Traditional target optimization may combine AI-based prioritization or rescoring with complementary structural tools such as protein structure prediction and molecular docking. Representative applications, strengths, limitations, and areas of use are summarized for each route.

The practical value of this synthesis tool lies in matching target discovery strategies to the biological maturity of candidate targets rather than favoring any specific computational method. Sequence and phenotypic data support variant prioritization, multi-omics analyses facilitate the discovery of previously unrecognized resistance determinants, and structural or network-based approaches help convert computational associations into experimentally testable targets [89]. Accordingly, data quality, biological plausibility, interpretability, and experimental validation determine whether an AI-prioritized target should advance beyond target selection. Once a target or resistance mechanism is sufficiently supported, the central question shifts from what should be targeted to how that information can be translated into an effective molecule or therapeutic regimen.

## The Applications and Evaluations of AI in the Design of Antibacterial Drug Molecules

Antibacterial molecular design translates validated resistance mechanisms and targets into optimized molecules or therapeutic strategies. AI-driven approaches can be broadly classified into mutation-tolerant design, multi-mechanism design, and combination-oriented regimen optimization. These strategies aim to maintain activity against target variants, overcome interconnected resistance pathways, or improve interactions between existing agents. AI can reduce the chemical and biological search space and prioritize candidates across multiple objectives, although experimental validation remains essential to confirm potency, selectivity, pharmacological properties, and translational potential.

### Mutation-tolerant molecular design for target structural variants

Resistance mutations can alter target conformation and ligand binding, limiting designs based on a single structure [90]. Mutation-tolerant design therefore emphasizes conformational coverage. Multi-conformation docking evaluates compound compatibility with altered targets, while generative models and RL expand chemical space and optimize multiple design objectives [91–93]. Their effectiveness depends on reliable structural information and experimental characterization of resistance mechanisms.

#### Multi-conformational computational design and AI-assisted prioritization

Resistance mutations can alter binding-site geometry and target conformational populations, limiting the reliability of single-structure screening. Conformational-ensemble approaches can better capture these changes, although studies of TEM β-lactamase variants show that population-weighted modeling may outperform conventional ensemble docking, indicating that more conformations do not necessarily improve prediction [94]. A complementary strategy is to prioritize compounds that retain activity across resistant variants, as demonstrated by allosteric and multi-variant dihydrofolate reductase (DHFR) inhibitors with activity against trimethoprim-resistant mutants [95,96]. However, mutation tolerance remains compound-dependent, as clinical imipenemase (IMP)-type metallo- β-lactamase variants can differentially affect inhibitor potency [97]. ML-guided screening has further extended variant-aware prioritization to mutant GyrA and ParC, but such predictions still require experimental validation [98].

#### Generative and reinforcement-learning optimization of mutation-tolerant molecules

Generative models and RL provide complementary strategies for expanding and optimizing candidate space in mutation-tolerant design. VAE-, GAN-, and diffusion-based approaches can generate novel molecules or peptide sequences beyond conventional screening libraries and prioritize candidates according to activity-related or structural constraints. Although direct applications to mutation-tolerant antibacterial small-molecule design remain limited, studies by Tucs et al. [99] and Lin et al. [100] demonstrated the feasibility of activity-guided GAN generation. More recent AMP-design strategies have extended this framework through different generative paradigms. Wang et al. [32] developed a latent diffusion pipeline coupled with molecular dynamics, generating peptides with lower sequence similarity and greater diversity than those produced by previously reported approaches, including GAN-based models. AMP-Designer further applied an LLM-based foundation model to support property-guided and strain-specific AMP generation, including settings with limited target-specific data [101]. For emerging drug-resistant pathogens, Yu et al. [102] combined meta-learning with RL to enable rapid adaptation from small pathogen-specific datasets. These approaches address different design needs rather than representing a single superior modeling strategy, with diffusion favoring broader sequence exploration, foundation models facilitating transferable and controllable design, and meta-learning providing an advantage when pathogen-specific data are limited. Importantly, computational prioritization does not necessarily translate into experimental activity, as illustrated by recent toxin-derived AMP screening in which only a limited subset of highly ranked candidates showed measurable antibacterial effects [103]. Thus, experimental validation remains essential when translating AI-generated or AI-prioritized peptides into viable antibacterial candidates. Importantly, generative models are more likely to yield experimentally tractable candidates when generation is constrained by synthetic accessibility or reaction feasibility, target- or pocket-specific interactions, and relevant physicochemical or off-target properties [22,104,105]. Without such constraints, they may primarily expand chemical space, generating structures that are difficult to synthesize or fail to show the predicted biological activity during experimental validation.

RL is particularly useful when multiple molecular properties must be optimized simultaneously. By incorporating antibacterial activity, binding performance, drug-likeness, synthetic accessibility, or pharmacological properties into reward functions, RL can iteratively prioritize candidates across competing design objectives [106,107]. AMPainter provides a representative example, using deep RL to optimize antimicrobial peptide sequences and substantially improve antibacterial activity [108]. Compared with generative models that emphasize candidate-space exploration, RL places greater emphasis on multi-objective optimization. However, both approaches depend strongly on the quality of training data, scoring models, and optimization criteria. Thus, computational novelty alone should not be considered evidence of a viable antibacterial candidate. Their application to mutation-tolerant antibacterial small molecules remains comparatively underdeveloped, and experimental validation is still required to confirm activity, toxicity, stability, and efficacy against resistant variants.

### Multi-mechanism synergistic molecular design for complex resistance mechanisms

Complex resistance phenotypes often involve multiple mechanisms, including enzymatic inactivation, efflux, altered permeability, and biofilm-associated tolerance [109]. Molecular design must therefore address interconnected resistance pathways rather than a single target. Current strategies include multi-target docking for structure-guided interaction analysis, multi-task learning for simultaneous efficacy and safety optimization, and fragment assembly for combining functional chemical motifs. Their applicability depends mainly on available mechanistic knowledge, structural information, and chemically tractable design elements.

#### Multi-target computational docking and AI-assisted prioritization

Multi-target computational screening can prioritize molecules engaging complementary bacterial pathways, but predicted binding does not always translate into balanced biological activity. ULD1/ULD2 and ketolide-quinolone hybrids provide relatively strong evidence because their dual mechanisms were experimentally supported [92]. By contrast, only subsets of designed gyrase/DHFR inhibitors retained dual-target activity, while AbaI/AbaR and GyrA/ParC studies remain largely computational [98,110,111]. Because most studies do not report the full proportion of prioritized candidates that remain active after validation, prediction-to-validation success rates are still difficult to estimate. Multi-target design should therefore be judged by balanced target engagement, phenotypic activity, and resistance suppression rather than computational scores alone.

#### Molecular generation based on multi-task learning

Multi-task learning is an AI modeling strategy suited to antibacterial design when activity across pathogens or mechanisms must be balanced with toxicity and developability [112]. Unlike multi-target docking, which is a non-AI structure-based tool for assessing ligand compatibility with predefined proteins, multi-task models learn shared representations across empirical labels and can support prediction, prioritization, or generation. This flexibility is also reflected in dsAMPGAN, which couples AMP recognition and generation within a dual-task framework and thus provides an alternative multi-task paradigm for AMP design. As summarized in Fig. 7, M3-CAD, APEX, and PolyCLOVER illustrate distinct applications of this principle rather than interchangeable success cases. M3-CAD emphasizes multimodal and mechanism-aware AMP generation, APEX prioritizes peptides at the proteome scale, and PolyCLOVER combines multi-task prediction with active learning to explore sparsely labeled polymer space [113–115].

**Fig. 7.**
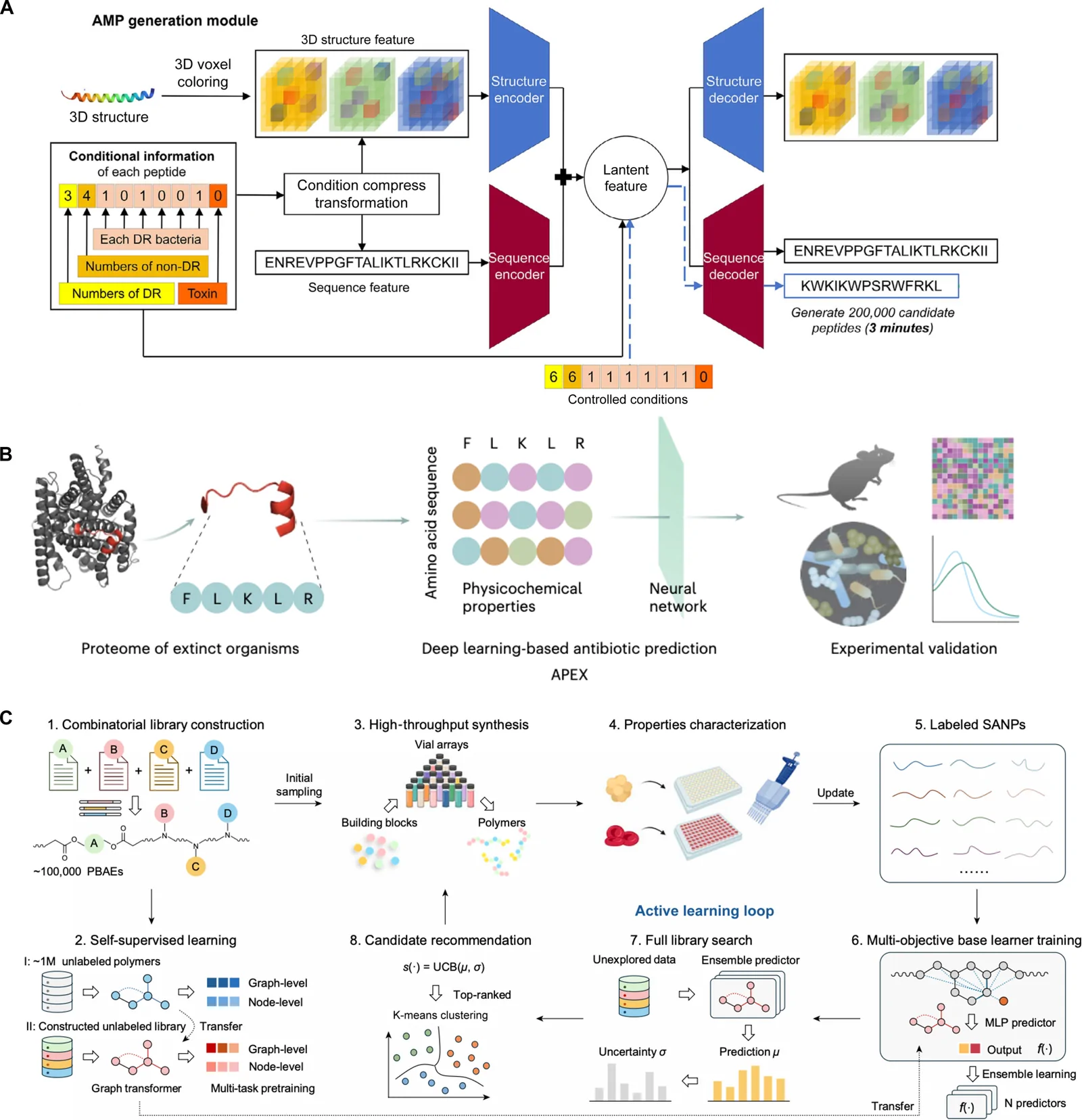
Multitask learning-guided generation of antibacterial peptides and polymeric antibiotics. (A) M3-CAD integrated peptide sequence, 3D structural features, antibacterial activity labels, and toxicity labels through a multimodal, multitask, and multilabel learning framework to generate multi-mechanism antibacterial peptides. Reproduced with permission from Ref. [114] under the Creative Commons Attribution 4.0 International License (CC BY 4.0). (B) APEX used proteome datasets from extinct organisms and multitask deep-learning models to screen short peptide sequences, generating antibiotic peptides active against clinically relevant pathogens. Reproduced with permission from Ref. [115] under the Creative Commons CC BY license. (C) PolyCLOVER constructed a combinatorial polymer library and combined self-supervised graph learning, ensemble prediction, and active learning to identify polymeric antibiotics with antibacterial activity and low hemolytic toxicity. Reproduced with permission from Ref. [113] under the Creative Commons CC-BY-NC-ND license.

Importantly, their experimental conversion rates are not equivalent. APEX yielded 41 active peptides among 69 synthesized candidates (59%), whereas M3-CAD reported in vitro antibacterial activity for 20 high-priority peptides but advanced only leading candidates to deeper mechanistic and in vivo validation [114,115]. PolyCLOVER experimentally tested successive candidate batches and ultimately identified 3 lead polymers from a library of approximately 100,000 structures [113]. These figures are not directly comparable because the studies used different preselection thresholds, molecular classes, activity criteria, and validation depths. Thus, AI performance should be judged not only by predictive metrics or successful lead examples but also by the proportion of prioritized candidates that retain multi-property activity after standardized experimental testing. Inconsistent reporting of this conversion currently limits robust cross-study estimates of prediction-to-validation success.

#### Fragment assembly design

Fragment assembly bridges fragment-based optimization and de novo generation by combining predefined functional motifs with optimized linkers to maintain structural compatibility and chemical feasibility [116]. Rule-based methods such as retrosynthetic combinatorial analysis procedure (RECAP) and breaking of retrosynthetically interesting chemical substructures (BRICS) support systematic fragment recombination, while generative models provide greater flexibility for linker and pocket-conditioned design. DeLinker [117], DiffLinker [118], and DiffSBDD [104] exemplify increasingly flexible approaches to linker or structure generation. Compared with unconstrained de novo generation, fragment assembly offers greater interpretability and a more restricted chemical space, but computationally compatible assemblies may still lose target engagement, synergy, or synthetic tractability. Therefore, fragment-based candidates require phenotypic and resistance validation before their predicted advantages can be established.

### Combination-oriented molecular design for optimizing antibacterial regimens

Combination-oriented design addresses antibacterial resistance by optimizing therapeutic regimens rather than developing a standalone antibacterial agent. AI can contribute at 3 complementary levels by predicting drug interactions, identifying biologically complementary pathways, and optimizing dosing under PK and PD constraints [119]. These capabilities support combination prioritization, mechanistic interpretation, and the design of clinically relevant drug exposure, respectively.

#### AI-assisted prediction and network-informed design of synergistic combinations

ML combination models integrate drug descriptors, target or omics information, resistance features, and measured interaction data to reduce the experimental screening space [120]. INDIGO and later chemogenomic or transcriptomic approaches show how biological context can improve prediction beyond chemical descriptors alone [121]. Yet, greater model complexity does not guarantee generalization across pathogens, concentration ranges, interaction metrics, or laboratory protocols; checkerboard, time-kill, and resistance-suppression experiments remain necessary before predicted synergy is considered actionable.

Network-informed models extend pairwise prediction by connecting drugs with targets, metabolic pathways, and resistance determinants. CALMA combines metabolic modeling with deep learning, while large interaction maps and chemical-genetic networks provide systems-level hypotheses for complementary combinations [122,123]. Such models can improve mechanistic plausibility, but incomplete pathway annotation and context-dependent bacterial physiology limit transfer between strains and infection conditions.

#### Design of combination therapy based on dose optimization algorithms

Whereas combination discovery addresses which agents should be paired, dose optimization determines how selected drugs should be administered to maximize bacterial clearance while limiting resistance emergence and toxicity. PK/PD models, bacterial dynamic models, population PK, and optimization algorithms can be integrated to evaluate drug ratios, dosing intervals, and exposure targets [124,125], making this approach especially relevant when effective combinations have been identified but their optimal therapeutic exposure remains uncertain. Smith et al. [126] demonstrated this strategy by integrating hollow-fiber infection data, PK/PD modeling, population PK, and a genetic algorithm to optimize meropenem and polymyxin B therapy against carbapenem-resistant *Acinetobacter baumannii*. Subsequent studies have extended this framework to multidrug-resistant Gram-negative pathogens and triple-drug regimens [127,128]. By coupling dynamic infection models with clinically achievable drug exposures, these approaches provide a translational pathway from combination efficacy assessment to optimized regimen design. Nevertheless, their clinical applicability depends on whether PK assumptions and dosing constraints adequately account for interindividual differences in organ function, disease severity, and treatment response variability.

### Integrated assessment and selection strategies for AI-driven antibacterial molecular design

Following upstream target selection, the downstream challenge is to determine how a sufficiently characterized target or resistance mechanism should be translated into an intervention [129]. Accordingly, Fig. 8 functions as a pragmatic synthesis and strategy-selection tool that summarizes 3 major design routes without introducing a separate theoretical model. Mutation-tolerant molecular design is particularly suitable when resistance mutations have been structurally defined and their effects on target interactions can be evaluated; multi-mechanism synergistic molecular design is more applicable to resistance phenotypes involving interconnected targets or pathways; whereas combination-oriented molecular design is preferable when existing antibacterial agents can be strategically integrated to enhance efficacy or limit resistance development.

**Fig. 8.**
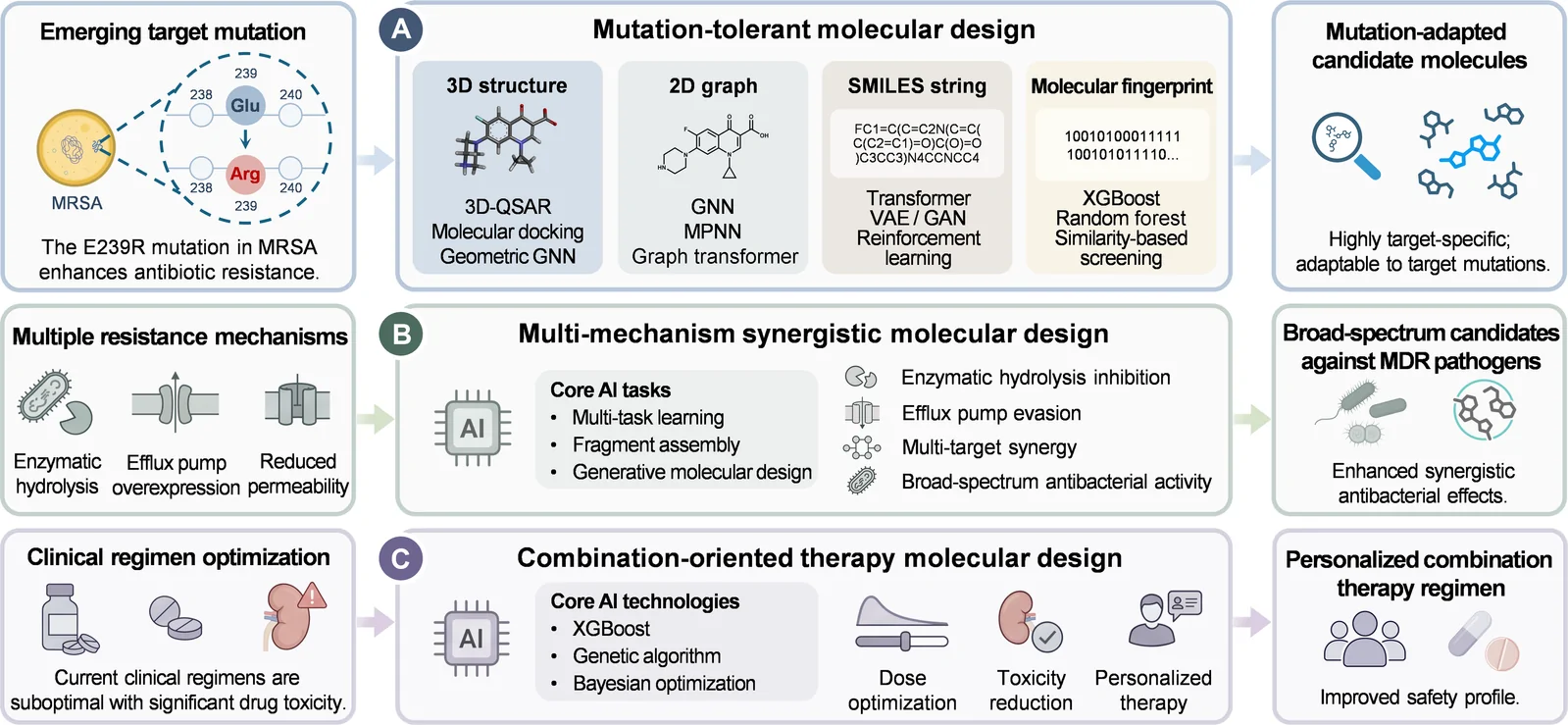
Integrated assessment and selection strategies for AI-assisted antibacterial molecular design. Three representative routes are shown, including mutation-tolerant molecular design (A), multi-mechanism synergistic molecular design (B), and combination-oriented therapy design (C). AI models are used for prediction, prioritization, generation, and regimen optimization, whereas molecular docking, 3D-QSAR, similarity-based screening, and related structure-based calculations serve as complementary non-AI computational tools within these workflows. These strategies support the development of mutation-adapted candidates, multi-mechanism antibacterial agents, and personalized combination regimens.

Unlike the upstream target-selection step, evaluation at this downstream stage is intervention-specific. Mutation-informed approaches depend largely on reliable structural and mutational information, multi-mechanism strategies require evidence linking multiple targets or biological activities, and combination or regimen optimization relies more extensively on quantitative drug interaction and PK/PD data. Structural characterization of PBP2a E239R demonstrates how mutation-specific insights can guide compound refinement [130,131], while the complex resistance profiles observed in CRAB and CRE provide a strong rationale for multi-mechanism intervention strategies [132]. Established therapeutic regimens, including polymyxin-based combinations, are more appropriately optimized through dose-exposure modeling approaches [133]. Across all 3 routes, computational success should be reported together with synthesis success, the fraction of candidates retaining predicted selectivity or multi-target activity, phenotypic potency across resistant strains, toxicity, PK/PD behavior, resistance emergence, and in vivo confirmation. Experimental validation varies substantially across representative AI-generated AMP studies. BroadAMP-GPT reported potent activity in 57% of tested candidates, AMPainter identified active AMPs in 6 of 10 de novo candidates, a ProtGPT2-based pipeline found potent activity in 4 of 9 candidates, and APEX yielded 41 active peptides among 69 synthesized candidates, whereas CVAE-BIO reported that 18.5% met the more stringent minimum inhibitory concentration (MIC) ≤10 μg/ml criterion [31,108,115,131,134]. These results are not directly comparable because candidate preselection and definitions of activity differ among studies, but they illustrate that computational prioritization does not uniformly translate into experimental success. Because these denominators are inconsistently reported, current literature probably overrepresents successful examples and does not yet support robust estimates of overall AI design success rates.

## Applications of AI in Antibacterial Efficacy Prediction

### AI-assisted prediction of antibacterial efficacy to mitigate resistance risk

Accurate antibacterial efficacy prediction links microbiological evidence with therapeutic decision-making and resistance management (Fig. 9) [135]. AI applications at this stage are best classified by decision scale, including rapid in vitro susceptibility assessment, patient-level outcome prediction, and resistance-evolution forecasting. Their clinical utility depends on matching input data to the intended task, from pathogen-level phenotypic or genotypic features to patient-specific exposure and longitudinal resistance surveillance.

**Fig. 9.**
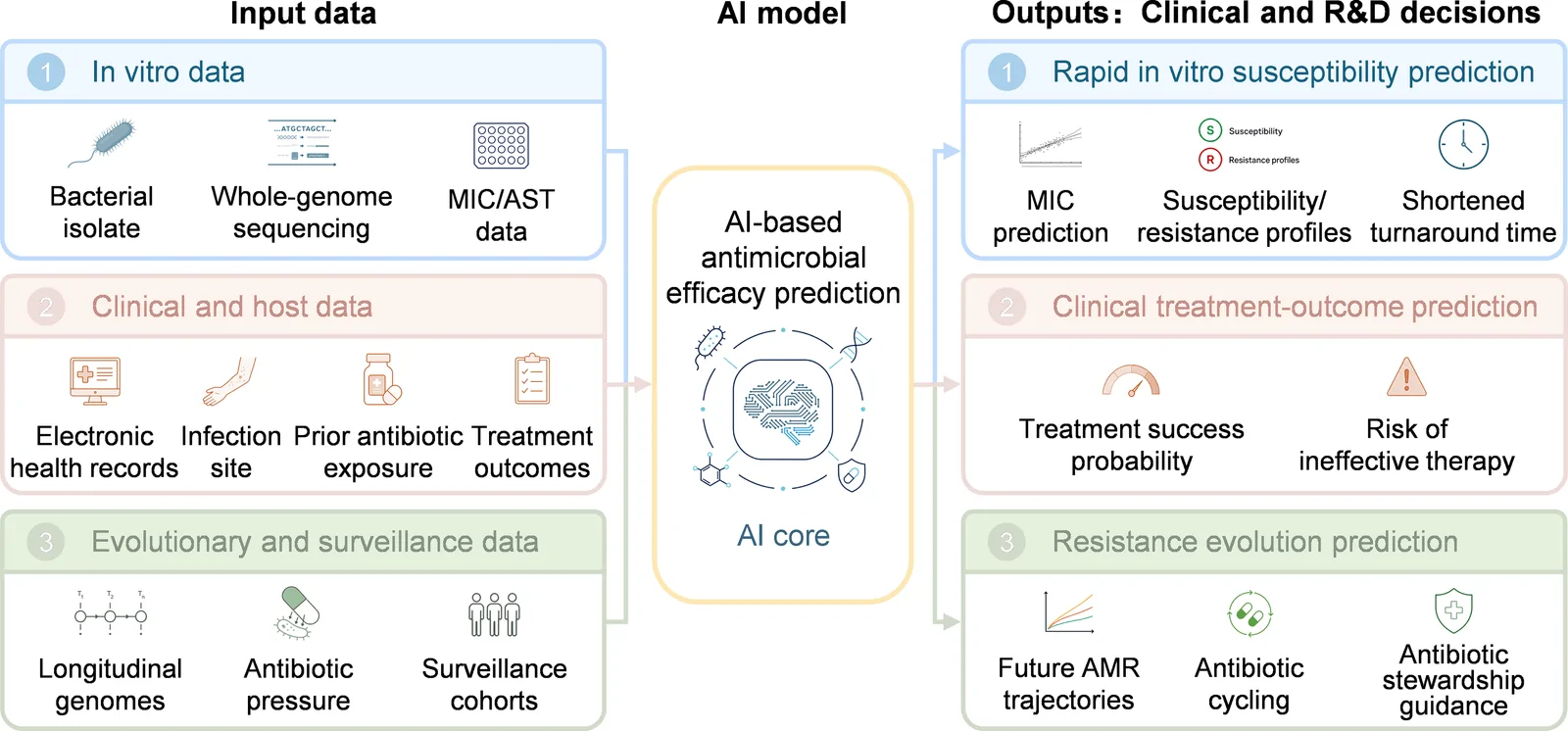
AI-assisted antibacterial efficacy prediction from input data to clinical and R&D decisions. AI models transform diverse input data into clinically and translationally relevant predictions for antibacterial efficacy assessment. By integrating experimental susceptibility data, patient-related clinical information, and resistance surveillance evidence, these models can support faster susceptibility assessment, more individualized treatment-outcome prediction, and forward-looking evaluation of resistance evolution, thereby providing decision support for antibacterial therapy, stewardship strategies, and antibacterial R&D.

#### In vitro efficacy prediction for antibacterial susceptibility assessment

Conventional antibacterial susceptibility testing often requires 48 to 72 h, highlighting the potential of AI-assisted approaches for accelerating susceptibility prediction [136,137]. Current evidence does not support a universally superior platform; instead, available approaches appear to provide complementary sources of information. For example, GenseqAMR predicted antibacterial susceptibility in *Klebsiella pneumoniae* using clinical shotgun metagenomic data, achieving area under the curve (AUC) values of 0.926 to 0.956 within 18.34 h [138], whereas matrix-assisted laser desorption/ionization time-of-flight mass spectrometry (MALDI-TOF)-based and nanomotion-assisted approaches infer susceptibility profiles from phenotypic signatures [139,140]. Genomic approaches provide broader mechanistic insights, whereas phenotypic methods can capture resistance mechanisms that remain unresolved by currently identified genetic determinants. Nevertheless, reported performance remains highly dependent on sample quality, pathogen diversity, and external validation. AI-based rapid prediction is therefore best positioned as a complementary tool that accelerates conventional susceptibility testing rather than replacing established diagnostic workflows.

#### Prediction of clinical treatment outcomes using in vitro and patient’s data

Clinical outcomes cannot be inferred from antibacterial susceptibility alone because treatment response is also influenced by drug exposure, infection site, host status, comorbidities, and treatment history. Accordingly, clinical outcome models integrate microbiological data with patient and therapeutic variables to support individualized decision-making. Rhodes et al. [141] showed that incorporating patient characteristics improved early risk stratification for methicillin-resistant *S. aureus* infection in hospitalized patients with community-acquired pneumonia. Subsequent studies using electronic health records, prior cultures, antibacterial exposure, and susceptibility profiles further demonstrate the value of incorporating clinical context rather than relying on isolated predictors [142–144]. However, these models are more sensitive than in vitro predictors to differences in patient populations, prescribing practices, and healthcare settings, making external and prospective validation essential. Their main value therefore lies in bridging microbiological susceptibility with real-world treatment conditions to identify therapies most likely to achieve meaningful clinical outcomes.

#### Prediction of antibiotic resistance evolution and emerging resistance mutations

Predicting resistance evolution extends AI-based assessment beyond current susceptibility by estimating how efficacy may change under future selective pressure [145]. Existing studies are more clearly understood according to decision scale rather than through individual case descriptions. Treatment-policy models address therapy-associated evolutionary dynamics [146], hospital-level models estimate institutional AMR risk [147], and genomic models examine dissemination or HGT at the population level [148,149]. Comparison across these scales shows that evolutionary prediction can support therapeutic planning, surveillance, and population-risk assessment, although the required data, time horizon, and interpretation differ substantially.

Compared with susceptibility testing and clinical outcome prediction, however, evolutionary modeling remains inherently more uncertain because resistance trajectories are shaped by dynamic selective pressures, transmission patterns, mutational events, and horizontal gene exchange. Accordingly, its practical utility is likely to be greatest in identifying emerging resistance risks and informing preventive strategies, whereas accurate long-term prediction remains limited by rare evolutionary events, incomplete surveillance coverage, and regional differences in sampling and epidemiological context.

### Integrated assessment of AI-based antibacterial efficacy prediction

AI-assisted efficacy prediction can instead be considered a sequential decision framework that extends from rapid susceptibility assessment to patient-specific treatment prediction and, ultimately, resistance risk forecasting [150]. The appropriate computational strategy is therefore determined by the clinical decision context and the type of information available at each stage. In vitro models emphasize rapid phenotypic or genotypic characterization, clinical prediction models incorporate host and treatment-related variables, and evolutionary models rely on longitudinal surveillance data to estimate emerging resistance risks.

Clinical-stage programs illustrate that AI can contribute at different points in antibacterial development, from early design decisions to later-stage analysis and optimization [151,152]. For LBP-EC01 and BX004, ML platforms were used mainly to support phage-cocktail selection and optimization rather than to generate phages de novo [153,154]. In comparison, AlphaFold2-based modeling of ibezapolstat contributed primarily to structural and mechanistic interpretation after the candidate had already been established [155]. P-PEAKS UTI and DANCE further extend this spectrum by using computational approaches for phage–host prediction, genomics-guided selection, and personalized matching (Table 2) [156,157]. These examples highlight the diverse roles of AI across the development process and underscore the importance of distinguishing design-defining applications from downstream or auxiliary support.

**Table 2.** Representative clinical-stage anti-infective programs with different levels of AI involvement in their development. Data source: United States National Clinical Trial official website (ClinicalTrials.gov, for all clinical trial registration details), published peer-reviewed medicinal chemistry literature, and official drug development documents from pharmaceutical enterprises (for specific artificial intelligence involvement information).

Project ID	Drug/program	Phases	Indication	Current status	AI contribution and role category
NCT04247542	Ibezapolstat	Phase II	*Clostridium difficile* infection	Completed	AlphaFold2-based structure prediction and docking were used to interpret PolC–ibezapolstat interactions and resistance-related residues after the drug had entered clinical development
NCT07513285	Ibezapolstat	Phase II	*Clostridium difficile* infection	Not yet recruiting	The available AI-assisted structural analyses relate to mechanistic interpretation of an established candidate
NCT04191148	LBP-EC01	Phase I	Lower urinary tract *E. coli* colonization	Completed	ML models were used to predict and select high-performing phage cocktails within a high-throughput platform, while computer vision supported phenotypic evaluation
NCT05488340	LBP-EC01	Phase II	Acute uncomplicated UTI caused by drug-resistant *E. coli*	Recruiting	ML models supported phage-cocktail prediction and selection, with robotics and computer vision used for high-throughput testing
101289070	P-PEAKS UTI	Phase II (planned)	Recurrent *E. coli* urinary tract infections	EU project ongoing (2026–2030); trial planned	AI-based phage–host prediction and phage activity clustering can support personalized phage selection and genomics-guided cocktail design based on the genome of the infecting strain.
NCT05177107	DANCE personalized phage therapy	Phase IIb	Diabetic foot osteomyelitis (*S. aureus*)	Terminated (minimum enrollment threshold reached)	Phage susceptibility testing and computational/bioinformatic matching were used to select existing phages for patient isolates
NCT05010577	BX004-A	Phase Ib/IIa	Chronic *P. aeruginosa* infection in cystic fibrosis	Completed	BiomX reported ML-based BOLT platform support for cocktail design, while peer-reviewed work documents computational cocktail selection and ML-assisted phage annotation

The translational outcomes of these programs also illustrate the current limitations of AI-assisted antibacterial development. Although several candidates have progressed into clinical evaluation, not all programs have advanced successfully. The BX004 phase 2b program, for example, was discontinued following unexpectedly high adverse-event rates and Data Monitoring Committee feedback, whereas the DANCE trial was terminated after reaching its minimum enrollment threshold. These outcomes cannot be attributed directly to AI, but they demonstrate that computationally guided design or selection does not necessarily translate into clinical success. Important bottlenecks also remain across different application settings. Susceptibility models can be affected by assay quality, phenotype definition, and clonal or site-specific bias, whereas clinical-outcome models depend on representative prospective and multicenter data. Reported AUC values, MIC changes, and other performance metrics are still dominated by retrospective, single-center, or in vitro evaluations, with prospective multicenter studies linked directly to clinical outcomes remaining limited. Resistance forecasting requires sufficiently longitudinal surveillance datasets, and molecular or phage optimization models may still inadequately capture safety, PK, host–environment interactions, and manufacturing constraints [158]. As of 2026, no antibacterial agent with both an AI-discovered target and an AI-designed therapeutic structure has received regulatory approval, and prospective multicenter evaluations linked directly to clinical outcomes remain limited. AI-based prediction and optimization should therefore be interpreted within the context of their validation setting and intended biological or clinical application.

## Core Challenges and Future Breakthrough Directions of AI-Empowered Antibacterial Drug and Antibiotic Design

### Current challenges limiting clinical translation of AI antibacterial discovery

Despite substantial progress in AI-assisted antibacterial discovery, translation remains limited. Because data heterogeneity, clonal bias, external-validation gaps, and in vitro–in vivo discrepancies are cross-cutting constraints, the discussion below focuses on how they affect evidence quality, reproducibility, and clinical implementation rather than repeatedly enumerating the same limitations. Reported AUCs, accuracies, MIC changes, and hit rates should therefore be interpreted in light of validation design, and no AI-discovered or AI-designed antibacterial agent has yet received regulatory approval [159,160].

#### Dual bottlenecks of data quality and quantity

Data limitations are better viewed as an evidence-quality problem than simply a shortage of samples. Differences in antimicrobial susceptibility testing (AST) methods, breakpoint definitions, sequencing workflows, annotation practices, and metadata can generate dataset shift, while over-representation of related strains can cause clonal leakage and inflate internal validation [54,161]. The priority is therefore not only to enlarge datasets but also to standardize metadata, separate related clones across training and test sets, and evaluate models in geographically and temporally independent cohorts. Prospective multicenter studies should additionally assess calibration, failure cases, and clinically meaningful outcomes rather than relying on internal numerical performance alone [161,162].

#### Core technical barriers

Technical barriers can be grouped into 2 main problems, including model interpretability and biological transferability. Deep-learning and generative models may rank or generate candidates without providing transparent mechanistic explanations [163,164], while in vitro-optimized outputs may fail in vivo because metabolism, tissue distribution, immune responses, infection microenvironments, and patient-specific PK are incompletely represented [165,166]. Docking and molecular dynamics can provide complementary structural checks but are neither substitutes for experimental validation nor AI models by themselves [167]. The priority is therefore to connect model outputs with mechanistic testing, PK/PD assessment, and iterative experimental feedback rather than adding more disconnected computational modules [134,165,167].

#### The last-mile obstacles to clinical translation

AI has substantially expanded target identification, virtual screening, molecular generation, and lead optimization, but these advances have not yet translated into demonstrably higher clinical success rates. Only a limited number of computationally discovered or designed antibacterial candidates have reached clinical trials, and no AI-discovered antibacterial drug has received regulatory approval [159,160]. Improvements in computational ranking, MIC prediction, or other surrogate endpoints therefore do not establish clinical benefit; AI-informed candidates or decision-support systems should demonstrate clinically meaningful added value against appropriate standard-of-care comparators. Regulatory expectations are also evolving around model credibility, data traceability, reproducibility, bias mitigation, and interpretability [168–170]. The U.S. Food and Drug Administration–European Medicines Agency (FDA-EMA) Guiding Principles of Good AI Practice emphasize human-centered and risk-based evaluation, clear context of use, data governance, performance assessment, lifecycle management, and transparent communication [169]. Computational outputs, including de novo molecules, predictive models, and optimized therapeutic strategies, should therefore show biological plausibility, experimental reproducibility, and clinical relevance. Limited mechanistic transparency may further complicate regulatory confidence [171,172]. In practical implementation, developers should define context of use, maintain traceable training and validation datasets, establish version control, quantify uncertainty, preserve audit records, and reproducibly link predictions to supporting experimental evidence.

Clinical translation also depends on population and implementation context. Performance may decline when infection-site biology, patient characteristics, prescribing practices, or resistance epidemiology differ across institutions [161,162]. Prospective multicenter studies should test added value beyond existing workflows, while implementation studies directly assess laboratory turnaround time, electronic health record (EHR) and AST interoperability, antimicrobial-stewardship integration, reimbursement, clinician acceptance, and performance drift [160,173]. These factors should be evaluated directly rather than inferred from retrospective computational performance.

#### Ethical, biosafety, privacy, and equity considerations

The application of AI in antibacterial discovery and resistance surveillance raises 3 distinct ethical and safety issues. First, models that combine pathogen genomics with molecular design capabilities could be misused to enhance virulence, resistance, or immune evasion, warranting risk-proportionate access controls, audit mechanisms, and biosafety and biosecurity review [174]. Second, genomic surveillance and EHR-linked AMR modeling may expose sensitive patient, community, or institutional information, supporting data minimization, secure governance, and privacy-preserving or federated analytical approaches where appropriate [175,176]. In addition, AI-derived drugs or decision tools may widen disparities if training datasets, clinical trials, manufacturing capacity, affordability, or implementation resources underrepresent low-resource settings [176,177]. Ethical safety should therefore be treated as a cross-cutting criterion from model development through clinical deployment rather than as a post hoc consideration.

### Future breakthrough directions

Addressing the current translational barriers will require coordinated advances in standardized antibacterial data infrastructure, regulatory oversight, translational validation, clinical trial design, and implementation frameworks [178]. As summarized in Fig. 10, these priorities encompass data infrastructure, model development, and clinical deployment, which together provide a foundation for the reliable translation of AI-enabled antibacterial research [179].

**Fig. 10.**
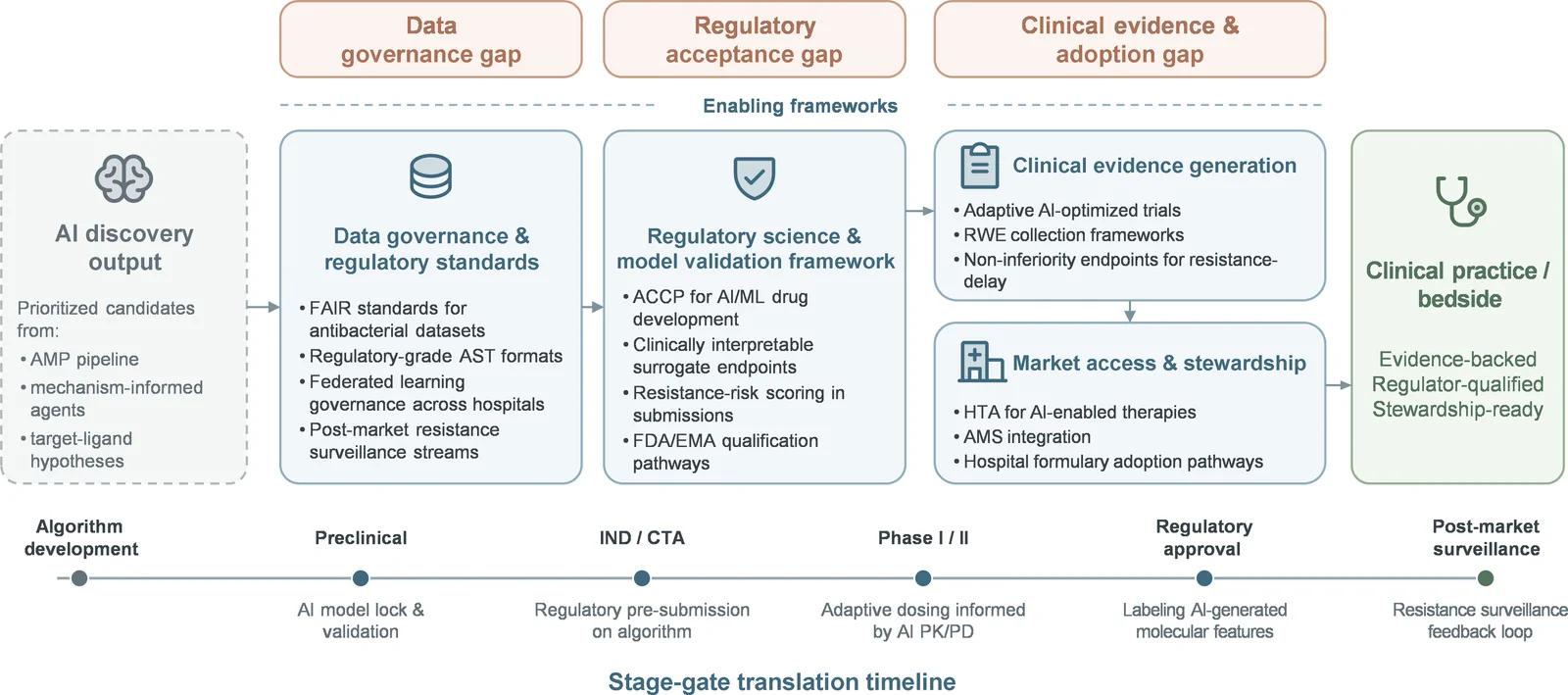
Future directions for translating AI-driven antibacterial discovery. Advancing AI-driven antibacterial discovery toward clinical translation requires coordinated progress across data, model, and clinical implementation dimensions. Current barriers include data bottlenecks, technical limitations, and translational barriers, which may reduce data reliability, model interpretability, and clinical applicability. Standardized data infrastructure, explainable resistance-aware AI, and clinical translation frameworks are proposed as key directions to address these limitations. These advances are expected to support a robust data ecosystem, interpretable drug design, and accelerated clinical translation of AI-enabled antibacterial agents.

Key unresolved questions include whether model performance remains robust across institutions, regions, pathogen populations, and time; whether in vitro improvements translate consistently into in vivo efficacy and patient benefit; how uncertainty, model updates, and algorithm modifications should enter regulatory evaluation; and whether AI-derived agents can be implemented safely, affordably, and equitably in routine practice. Future efforts should prioritize evidence robustness and clinical utility over incremental gains in benchmark performance.

#### Building standardized and integrated antibacterial data infrastructures

A standardized and integrated antibacterial data infrastructure is essential for robust and generalizable AI. International organizations, including World Health Organization (WHO), European Centre for Disease Prevention and Control (ECDC), Centers for Disease Control and Prevention (CDC), and China CDC, could support harmonized protocols integrating molecular, phenotypic, exposure-related, and clinical outcome data. Core requirements include sequencing quality control, AST procedures, infection-site annotation, treatment outcomes, and adverse-event documentation. Standardized AST methods together with WHO Global Antimicrobial Resistance and Use Surveillance System (GLASS) could improve cross-laboratory comparability, consistent reporting, and AI-compatible data exchange [180,181]. Global repositories should represent susceptible and resistant strains, resistance determinants, genetic variants, biofilm-associated phenotypes, and treatment outcomes while recognizing high-quality data contributors. Federated learning can enable collaborative model development across institutions without transferring raw patient or institutional data [175,182]. Beyond conventional resistance labels, next-generation datasets should capture intermediate phenotypes, biofilm responses, and temporal changes under antibiotic exposure. Rapid phenotypic AST technologies can provide dynamic growth and response features beyond static endpoints [183], enabling models to move from binary susceptibility classification toward prediction of resistance trajectories and treatment responses.

#### Developing interpretable, biologically integrated, and resistance-aware AI models

Future AI models should combine explainability, biological knowledge, and resistance-aware modeling across antibacterial discovery workflows. Explainable artificial intelligence (XAI) should be incorporated into molecular generation, activity prediction, and clinical decision support to improve interpretability [163]. Attention-based graph models, feature attribution, molecular fragment analysis, and 3-dimensional (3D) visualization can help identify functionally important chemical groups, binding regions, and mutation-sensitive interactions [164], while interpretable modeling can relate resistance determinants and strain characteristics to AMR predictions [171]. In parallel, models should move beyond general antibacterial activity and incorporate resistance mechanisms as design principles [184]. Mechanism-oriented approaches can integrate target mutations, enzymatic inactivation, efflux, biofilm-associated tolerance, membrane permeability, persistence, host–pathogen interactions, and microbiome effects. Multi-objective optimization should also balance potency, target selectivity, resistance suppression, biofilm penetration, PK, toxicity, and synthetic feasibility, supporting mutation-tolerant, biofilm-penetrating, or resistance-reversing agents against difficult pathogens, including multidrug-resistant *A. baumannii* and *P. aeruginosa* [114].

#### Establishing an integrated translational validation and clinical evaluation framework

A standardized translational framework should bridge computational predictions with experimental validation, animal efficacy, and clinical outcomes. Multiscale approaches incorporating infection-site biology, host PK, physiologically based pharmacokinetic/pharmacodynamic (PBPK/PD) modeling, organ-on-chip systems, and humanized infection models may improve translational relevance [185,186]. Computational predictions should be iteratively refined using orthogonal experimental evidence, including activity and safety assessments where appropriate [134,167]. Before clinical advancement, candidates should also be independently evaluated across laboratories, diverse strain collections, relevant infection models, resistance-evolution conditions, and clinically realistic dosing regimens.

As candidates enter clinical development, AI may support patient stratification, trial optimization, and real-world evidence generation [160,179]. However, prospective and external validation remain essential before such models can reliably guide clinical decisions. Multicenter networks and federated-learning frameworks may further enable distributed data analysis while improving privacy protection and external validation [175,182]. Harmonized AST and surveillance standards [180,181], together with PK/PD and clinical outcome data, can further strengthen cross-site comparability.

Regulatory planning should therefore begin early and continue throughout development. Current guidance emphasizes data governance, risk-based performance assessment, model life-cycle management, human oversight, and performance monitoring [168–170]. Clinical implementation should additionally consider cybersecurity, interoperability, and compatibility with healthcare workflows [169,170,179].

#### A staged roadmap from discovery to real-world implementation

A practical roadmap for translating AI-enabled antibacterial discovery can be organized into 4 iterative stages. First, data readiness requires representative, standardized datasets with documented provenance, quantified uncertainty, and predefined external test cohorts to limit data leakage and clonal bias [54]. Second, translational validation requires independent evaluation across laboratories, diverse strain panels, resistance-evolution assays, relevant PK/PD and toxicity studies, and appropriate in vivo models [187]. Third, clinical evidence should come from prospective, preferably multicenter studies with predefined endpoints, subgroup analyses, and direct assessment of added value beyond conventional discovery or clinical workflows [188,189].

The final stage addresses implementation and lifecycle governance through regulatory documentation, interoperable deployment, antimicrobial-stewardship integration, economic evaluation, equitable access, model version control, and post-deployment surveillance. Advancement should follow predefined decision criteria, so strong internal metrics alone cannot support clinical translation without independent validation and demonstrated clinical utility.

## Conclusion

The global spread of bacterial resistance has become one of the defining public health challenges of our time, with AMR now responsible for a substantial share of deaths worldwide [182]. Traditional antimicrobial discovery is increasingly constrained by target homogeneity, limited chemical innovation, and insufficient consideration of resistance evolution. By harnessing large-scale data and uncovering hidden biological patterns, AI opens new avenues for reshaping antibacterial drug discovery [190].

This review has traced how AI is being applied across antimicrobial and antibiotic design. In target discovery, it can bring together multi-omics, genomic, and phenotypic data to pinpoint regulatory nodes tied to resistance, virulence, or biofilm formation, widening the pool of targets worth pursuing. In molecular design, deep learning and generative models open up new regions of chemical and peptide space, helping surface antibiotics and antimicrobial peptides with genuinely novel structures [191]. Recent studies further demonstrate that explainable deep learning can identify antibacterial structural classes with improved mechanistic insight, while generative AI can accelerate the discovery of antimicrobial peptides against multidrug-resistant bacteria [12,182].

In efficacy prediction and resistance control, AI supports susceptibility prediction, candidate prioritization, early warning of resistance evolution, and the rational design of combination or resistance-reversal strategies. Advances like these can make early-stage screening more efficient and feed useful evidence into precision antimicrobial therapy. Nevertheless, several barriers remain, including heterogeneous data quality, limited model interpretability, insufficient in vivo validation, and incomplete clinical integration. Building on the pragmatic synthesis tools and the staged roadmap for clinical translation outlined in this review, we suggest a gradual shift toward a more resistance-aware, AI-assisted research and development (R&D) model that complements conventional strategies, supported by more standardized data infrastructures, more interpretable AI platforms, and better-coordinated translational and regulatory pathways. If these efforts can be advanced through cross-sector collaboration, such a model may help accelerate the development of new anti-resistance agents and support a more proactive response to AMR, although its real-world value will need to be confirmed through further clinical and translational work.

## References

[1] GBD 2021 Antimicrobial Resistance Collaborators. Global burden of bacterial antimicrobial resistance 1990–2021: A systematic analysis with forecasts to 2050. Lancet. 2024;404(10459):1199–1226. 10.1016/S0140-6736(24)01867-1 PMC11718157 39299261

[2] Abdallah EM, Alhudhaibi AM, Dahab M, Al Noman A, Sharma PD, Taha TH, Nawaz M. The WHO priority list of antibiotic-resistant bacteria: Challenges and opportunities for next-generation antimicrobial development. Front Pharmacol. 2026;17:1699987. 10.3389/fphar.2026.1699987 PMC13106162 42038316

[3] Ribeiro Da Cunha B, Fonseca LP, Calado CRC. Antibiotic discovery: Where have we come from, where do we go? Antibiotics. 2019;8(2):45. 10.3390/antibiotics8020045 PMC6627412 31022923

[4] Melo MC, Maasch JR, De La Fuente-Nunez C. Accelerating antibiotic discovery through artificial intelligence. Commun Biol. 2021;4(1):1050. 10.1038/s42003-021-02586-0 PMC8429579 34504303

[5] Lluka T, Stokes JM. Antibiotic discovery in the artificial intelligence era. Ann N Y Acad Sci. 2023;1519(1):74–93. 10.1111/nyas.14930 36447334

[6] Cesaro A, Hoffman SC, Das P, De La Fuente-Nunez C. Challenges and applications of artificial intelligence in infectious diseases and antimicrobial resistance. NPJ Antimicrob Resist. 2025;3(1):2. 10.1038/s44259-024-00068-x PMC11721440 39843587

[7] Wong F, Zheng EJ, Valeri JA, Donghia NM, Anahtar MN, Omori S, Li A, Cubillos-Ruiz A, Krishnan A, Jin W, et al. Discovery of a structural class of antibiotics with explainable deep learning. Nature. 2024;626(7997):177–185. 10.1038/s41586-023-06887-8 PMC10866013 38123686

[8] Wong F, Krishnan A, Zheng EJ, Stärk H, Manson AL, Earl AM, Jaakkola T, Collins JJ. Benchmarking AlphaFold-enabled molecular docking predictions for antibiotic discovery. Mol Syst Biol. 2022;18(9): Article e11081. 10.15252/msb.202211081 PMC9446081 36065847

[9] Papp-Wallace KM, Endimiani A, Taracila MA, Bonomo RA. Carbapenems: Past, present, and future. Antimicrob Agents Chemother. 2011;55(11):4943–4960. 10.1128/AAC.00296-11 PMC3195018 21859938

[10] Yekani M, Ghanbari H, Azimi S, Milani ES, Hajibonabi A, Alizadeh N, Mir SM, Fard JK, Memar MY, Hosseinpour R. β-Lactamase inhibitors: A promising strategy for restoring carbapenem effectiveness. Eur J Pharmacol. 2025;1007: Article 178226. 10.1016/j.ejphar.2025.178226 41062009

[11] Yigit H, Queenan AM, Anderson GJ, Domenech-Sanchez A, Biddle JW, Steward CD, Alberti S, Bush K, Tenover FC. Novel carbapenem-hydrolyzing β-lactamase, KPC-1, from a carbapenem-resistant strain of Klebsiella pneumoniae. Antimicrob Agents Chemother. 2001;45(4):1151–1161. 10.1128/AAC.45.4.1151-1161.2001 PMC90438 11257029

[12] Rather MA, Gupta K, Mandal M. Microbial biofilm: Formation, architecture, antibiotic resistance, and control strategies. Braz J Microbiol. 2021;52(4):1701–1718. 10.1007/s42770-021-00624-x PMC8578483 34558029

[13] Mendes RE, Tsakris A, Sader HS, Jones RN, Biek D, Mcghee P, Appelbaum PC, Kosowska-Shick K. Characterization of methicillin-resistant Staphylococcus aureus displaying increased MICs of ceftaroline. J Antimicrob Chemother. 2012;67(6):1321–1324. 10.1093/jac/dks069 22398650

[14] Roy S, Junghare V, Dutta S, Hazra S, Basu S. Differential binding of carbapenems with the AdeABC efflux pump and modulation of the expression of AdeB linked to novel mutations within two-component system AdeRS in carbapenem-resistant Acinetobacter baumannii. MSystems. 2022;7(4): Article e0021722. 10.1128/msystems.00217-22 PMC9426577 35735748

[15] Xie L, Li J, Peng Q, Liu X, Lin F, Dai X, Ling B. Contribution of RND superfamily multidrug efflux pumps AdeABC, AdeFGH, and AdeIJK to antimicrobial resistance and virulence factors in multidrug-resistant Acinetobacter baumannii AYE. Antimicrob Agents Chemother. 2025;69(7):e01858-24. 10.1128/aac.01858-24 PMC12217454 40407309

[16] Kavanaugh LG, Hinrichsen ME, Dunham CM, Conn GL. Regulation, structure, and activity of the Pseudomonas aeruginosa MexXY efflux system. Antimicrob Agents Chemother. 2025;69(5): Article e01824. 10.1128/aac.01825-24 PMC12057347 40192483

[17] Flemming H-C, Van Hullebusch ED, Neu TR, Nielsen PH, Seviour T, Stoodley P, Wingender J, Wuertz S. The biofilm matrix: Multitasking in a shared space. Nat Rev Microbiol. 2023;21(2):70–86. 10.1038/s41579-022-00791-0 36127518

[18] Kavvas ES, Catoiu E, Mih N, Yurkovich JT, Seif Y, Dillon N, Heckmann D, Anand A, Yang L, Nizet V. Machine learning and structural analysis of Mycobacterium tuberculosis pan-genome identifies genetic signatures of antibiotic resistance. Nat Commun. 2018;9(1):4306. 10.1038/s41467-018-06634-y PMC6193043 30333483

[19] Li G, Bai P, Chen J, Liang C. Identifying virulence factors using graph transformer autoencoder with ESMFold-predicted structures. Comput Biol Med. 2024;170: Article 108062. 10.1016/j.compbiomed.2024.108062 38308869

[20] Yang B, Yang H, Liang J, Chen J, Wang C, Wang Y, Wang J, Luo W, Deng T, Guo J. A review on the screening methods for the discovery of natural antimicrobial peptides. J Pharm Anal. 2025;15(1): Article 101046. 10.1016/j.jpha.2024.101046 PMC11780100 39885972

[21] Li Q, Xu Z, Zhu Y, Zhou W, Guan M, Zhao S, Liu M, Liu B, Liu J. DTBind: A mechanism-driven deep learning framework for accurate prediction of drug-target molecular recognition. Research. 2025;8:1022. 10.34133/research.1022 PMC12669800 41341719

[22] Swanson K, Liu G, Catacutan DB, Arnold A, Zou J, Stokes JM. Generative AI for designing and validating easily synthesizable and structurally novel antibiotics. Nat Mach Intell. 2024;6(3):338–353.

[23] Swanson K, Liu G, Catacutan DB, Mclellan S, Arnold A, Tu MM, Brown ED, Zou J, Stokes JM. SyntheMol-RL: A flexible reinforcement learning framework for designing easily synthesizable antibiotics. Mol Syst Biol. 2026;22(6):833–867. 10.1038/s44320-026-00206-9 PMC13230741 42026234

[24] Wen T, Cai X, Li J. Graph neural networks vs. traditional QSAR: A comprehensive comparison for multi-label molecular odor prediction. Molecules. 2025;30(23):4605. 10.3390/molecules30234605 PMC12693603 41375203

[25] Krishnan A, Anahtar MN, Valeri JA, Jin W, Donghia NM, Sieben L, Luttens A, Zhang Y, Modaresi SM, Hennes A, et al. A generative deep learning approach to de novo antibiotic design. Cell. 2025;188(21):5962–5979.e22. 10.1016/j.cell.2025.07.033 PMC12790861 40816267

[26] Gröschel MI, Owens M, Freschi L, Vargas R Jr, Marin MG, Phelan J, Iqbal Z, Dixit A, Farhat MR. GenTB: A user-friendly genome-based predictor for tuberculosis resistance powered by machine learning. Genome Med. 2021;13(1):138. 10.1186/s13073-021-00953-4 PMC8407037 34461978

[27] Li Y, Xu Z, Han W, Cao H, Umarov R, Yan A, Fan M, Chen H, Duarte CM, Li L, et al. HMD-ARG: Hierarchical multi-task deep learning for annotating antibiotic resistance genes. Microbiome. 2021;9(1):40. 10.1186/s40168-021-01002-3 PMC7871585 33557954

[28] López-Cortés XA, Manríquez-Troncoso JM, Hernández-García R, Peralta D. MSDeepAMR: Antimicrobial resistance prediction based on deep neural networks and transfer learning. Front Microbiol. 2024;15:1361795. 10.3389/fmicb.2024.1361795 PMC11062410 38694798

[29] Lassen JK, Villesen P. End-to-end deep learning explains antimicrobial resistance in peak-picking-free MALDI-MS data. Anal Chem. 2025;97(5):2795–2800. 10.1021/acs.analchem.4c05113 PMC11824369 39893590

[30] Johnson WC, Alivisatos A, Smith TC, Van N, Soni V, Wallach JB, Clark NA, Fitzgerald TA, Whiteley JJ, Tan S, et al. Integration of multi-modal measurements identifies critical mechanisms of tuberculosis drug action. Cell Syst. 2025;16(8): Article 101348. 10.1016/j.cels.2025.101348 PMC12365861 40738114

[31] Li Y, Xu X, Zhang X, Xu Z, Zhao J, Zhu R, Wang Z, Ran W, Zhao W, Yan N, et al. BroadAMP-GPT: AI-Driven generation of broad-spectrum antimicrobial peptides for combating multidrug-resistant ESKAPE pathogens. Gut Microbes. 2025;17(1):2523811. 10.1080/19490976.2025.2523811 PMC12203862 40568788

[32] Wang Y, Song M, Liu F, Liang Z, Hong R, Dong Y, Luan H, Fu X, Yuan W, Fang W, et al. Artificial intelligence using a latent diffusion model enables the generation of diverse and potent antimicrobial peptides. Sci Adv. 2025;11(6): Article eadp7171. 10.1126/sciadv.adp7171 PMC11797553 39908380

[33] Gómez-Bombarelli R, Wei JN, Duvenaud D, Hernández-Lobato JM, Sánchez-Lengeling B, Sheberla D, Aguilera-Iparraguirre J, Hirzel TD, Adams RP, Aspuru-Guzik A. Automatic chemical design using a data-driven continuous representation of molecules. ACS Cent Sci. 2018;4(2):268–276. 10.1021/acscentsci.7b00572 PMC5833007 29532027

[34] Zervou MA, Doutsi E, Pantazis Y, Tsakalides P. De novo antimicrobial peptide design with feedback generative adversarial networks. Int J Mol Sci. 2024;25(10):5506. 10.3390/ijms25105506 PMC11122239 38791544

[35] Yagimoto K, Hosoda S, Sato M, Hamada M. Prediction of antibiotic resistance mechanisms using a protein language model. Bioinformatics. 2024;40(10): Article btae550. 10.1093/bioinformatics/btae550 PMC11464418 39254573

[36] Liu Z, Lei J, Zhang X, Yin J, Zhang Y, Lei K, Geng Y, Huang L, Han Q, He A. Evolution of ceftazidime-avibactam resistance driven by variation in bla KPC-2 to bla KPC-190 during treatment of ST11-K64 hypervirulent Klebsiella pneumoniae. Front Cell Infect Microbiol. 2025;15:1607127. 10.3389/fcimb.2025.1607127 PMC12179193 40546282

[37] Ünlü A, Çevrim E, Yiğit MG, Sarıgün A, Çelikbilek H, Bayram O, Kahraman DC, Olğaç A, Rifaioglu AS, Banoğlu E, et al. Target-specific de novo design of drug candidate molecules with graph-transformer-based generative adversarial networks. Nat Mach Intell. 2025;7(9):1524–1540.

[38] Lin S, Tu C, Hu R, Wang H, Dong Z, Luo H, Kuang L, Wang T, Wang L, Zhao Z, et al. Dr. Kinase: Predicting the drug-resistance hotspots of protein kinases. Nucleic Acids Res. 2025;53(W1):W258–W265. 10.1093/nar/gkaf366 PMC12230729 40308214

[39] Boudza R, Bounou S, Segura-Garcia J, Moukadiri I, Maicas S. Artificial intelligence as a catalyst for antimicrobial discovery: From predictive models to de novo design. Microorganisms. 2026;14(2):394. 10.3390/microorganisms14020394 PMC12943268 41753681

[40] Van Den Broek RL, Patel S, Van Westen GJ, Jespers W, Sherman W. In search of beautiful molecules: A perspective on generative modeling for drug design. J Chem Inf Model. 2025;65(18):9383–9397. 10.1021/acs.jcim.5c01203 PMC12458704 40893044

[41] Ardila CM, Yadalam PK, González-Arroyave D. Integrating whole genome sequencing and machine learning for predicting antimicrobial resistance in critical pathogens: A systematic review of antimicrobial susceptibility tests. PeerJ. 2024;12: Article e18213. 10.7717/peerj.18213 PMC11470768 39399439

[42] Scardino V, Di Filippo JI, Cavasotto CN. How good are AlphaFold models for docking-based virtual screening? IScience. 2023;26(1): Article 105920. 10.1016/j.isci.2022.105920 PMC9852548 36686396

[43] Szymczak P, Zarzecki W, Wang J, Duan Y, Wang J, Coelho LP, De La Fuente-Nunez C, Szczurek E. AI-driven antimicrobial peptide discovery: Mining and generation. Acc Chem Res. 2025;58(12):1831. 10.1021/acs.accounts.0c00594 PMC12177927 40459283

[44] Santos-Júnior CD, Torres MDT, Duan Y, Rodríguez Del Río Á, Schmidt TSB, Chong H, Fullam A, Kuhn M, Zhu C, Houseman A, et al. Discovery of antimicrobial peptides in the global microbiome with machine learning. Cell. 2024;187(14):3761–3778.e16. 10.1016/j.cell.2024.05.013 PMC11666328 38843834

[45] Karger E, Kureljusic M. Using artificial intelligence for drug discovery: A bibliometric study and future research agenda. Pharmaceuticals. 2022;15(12):1492. 10.3390/ph15121492 PMC9785219 36558943

[46] Sha Sajadieh, Fattorini L, Perrault R, Gil Y, Parli V, Santarlasci L, Pava J, Maslej N, Altman R, Brynjolfsson E, et al. The AI Index 2026 Annual Report. Stanford, CA: Institute for Human-Centered AI, Stanford University; 2026. arXiv. https://doi.org/10.48550/arXiv.2606.15708.

[47] Chen S, Yu M, Xu M, Decherchi S, Taracila MA, Hujer AM, Bethel CR, Bonomo RA, Haider S. Structural and kinetic basis for the rational design of next-generation β-lactamase inhibitors. J Med Chem. 2026;69(6):7262–7275. 10.1021/acs.jmedchem.5c03315 PMC13036776 41838618

[48] Blake KS, Kumar H, Loganathan A, Williford EE, Diorio-Toth L, Xue Y-P, Tang WK, Campbell TP, Chong DD, Angtuaco S, et al. Sequence-structure-function characterization of the emerging tetracycline destructase family of antibiotic resistance enzymes. Commun Biol. 2024;7(1):336. 10.1038/s42003-024-06023-w PMC10944477 38493211

[49] Lakin SM, Kuhnle A, Alipanahi B, Noyes NR, Dean C, Muggli M, Raymond R, Abdo Z, Prosperi M, Belk KE, et al. Hierarchical Hidden Markov models enable accurate and diverse detection of antimicrobial resistance sequences. Commun Biol. 2019;2(1):294. 10.1038/s42003-019-0545-9 PMC6684577 31396574

[50] Pal A, Mohanty D. Machine learning-based approach for identification of new resistance associated mutations from whole genome sequences of Mycobacterium tuberculosis. Bioinform Adv. 2025;5(1): Article vbaf050. 10.1093/bioadv/vbaf050 PMC11930343 40125545

[51] Pearcy N, Hu Y, Baker M, Maciel-Guerra A, Xue N, Wang W, Kaler J, Peng Z, Li F, Dottorini T. Genome-scale metabolic models and machine learning reveal genetic determinants of antibiotic resistance in Escherichia coli and unravel the underlying metabolic adaptation mechanisms. Msystems. 2021;6(4): Article e0091320. 10.1128/mSystems.00913-20 PMC8409726 34342537

[52] Mishra B, Basu A, Shehadeh F, Felix L, Kollala SS, Chhonker YS, Naik MT, Dellis C, Zhang L, Ganesan N, Article et al. Antimicrobial peptide developed with machine learning sequence optimization targets drug resistant Staphylococcus aureus in mice. J Clin Invest. 2025;135(12): Article e185430. 10.1172/JCI185430 PMC12165799 40261713

[53] Chen ML, Doddi A, Royer J, Freschi L, Schito M, Ezewudo M, Kohane IS, Beam A, Farhat M. Beyond multidrug resistance: Leveraging rare variants with machine and statistical learning models in Mycobacterium tuberculosis resistance prediction. EBioMedicine. 2019;43:356–369. 10.1016/j.ebiom.2019.04.016 PMC6557804 31047860

[54] Yu Y, Wheeler NE, Barquist L. Biased sampling driven by bacterial population structure confounds machine learning prediction of antimicrobial resistance. PLOS Biol. 2025;23(12): Article e3003539. 10.1371/journal.pbio.3003539 PMC12707637 41401143

[55] Lyu J, Kapolka N, Gumpper R, Alon A, Wang L, Jain MK, Barros-Álvarez X, Sakamoto K, Kim Y, Diberto J, et al. AlphaFold2 structures guide prospective ligand discovery. Science. 2024;384(6702): Article eadn6354. 10.1126/science.adn6354 PMC11253030 38753765

[56] Díaz-Holguín A, Saarinen M, Vo DD, Sturchio A, Branzell N, Cabeza De Vaca I, Hu H, Mitjavila-Domènech N, Lindqvist A, Baranczewski P, et al. AlphaFold accelerated discovery of psychotropic agonists targeting the trace amine–associated receptor 1. Sci Adv. 2024;10(32): Article eadn1524. 10.1126/sciadv.adn1524 PMC11305387 39110804

[57] Rahman S, Das AK. Integrated multi-omics, virtual screening and molecular docking analysis of methicillin-resistant Staphylococcus aureus USA300 for the identification of potential therapeutic targets: An in-silico approach. Int J Pept Res Ther. 2021;27(4):2735–2755. 10.1007/s10989-021-10287-9 PMC8446483 34548853

[58] Joshi T, Vijayakumar S, Ghosh S, Mathpal S, Ramaiah S, Anbarasu A. Identifying novel therapeutics for the resistant mutant “F533L” in PBP3 of Pseudomonas aeruginosa using ML techniques. ACS Omega. 2024;9(26):28046–28060. 10.1021/acsomega.4c00929 PMC11223260 38973840

[59] Jambrich MA, Tusnady GE, Dobson L. How AlphaFold2 shaped the structural coverage of the human transmembrane proteome. Sci Rep. 2023;13(1):20283. 10.1038/s41598-023-47204-7 PMC10662385 37985809

[60] Lyu J, Irwin JJ, Shoichet BK. Modeling the expansion of virtual screening libraries. Nat Chem Biol. 2023;19(6):712–718. 10.1038/s41589-022-01234-w PMC10243288 36646956

[61] Sadybekov AA, Sadybekov AV, Liu Y, Iliopoulos-Tsoutsouvas C, Huang XP, Pickett J, Houser B, Patel N, Tran NK, Tong F, et al. Synthon-based ligand discovery in virtual libraries of over 11 billion compounds. Nature. 2022;601(7893):452–459. 10.1038/s41586-021-04220-9 PMC9763054 34912117

[62] Bilal H, Khan MN, Khan S, Shafiq M, Fang W, Khan RU, Rahman MU, Li X, Lv Q-L, Xu B. The role of artificial intelligence and machine learning in predicting and combating antimicrobial resistance. Comput Struct Biotechnol J. 2025;27:423–439. 10.1016/j.csbj.2025.01.006 PMC11791014 39906157

[63] Argelaguet R, Arnol D, Bredikhin D, Deloro Y, Velten B, Marioni JC, Stegle O. MOFA+: A statistical framework for comprehensive integration of multi-modal single-cell data. Genome Biol. 2020;21(1):111. 10.1186/s13059-020-02015-1 PMC7212577 32393329

[64] Kim M, Rai N, Zorraquino V, Tagkopoulos I. Multi-omics integration accurately predicts cellular state in unexplored conditions for Escherichia coli. Nat Commun. 2016;7(1):13090. 10.1038/ncomms13090 PMC5059772 27713404

[65] Hyun JC, Monk JM, Szubin R, Hefner Y, Palsson BO. Global pathogenomic analysis identifies known and candidate genetic antimicrobial resistance determinants in twelve species. Nat Commun. 2023;14(1):7690. 10.1038/s41467-023-43549-9 PMC10673929 38001096

[66] Blattman SB, Jiang W, Oikonomou P, Tavazoie S. Prokaryotic single-cell RNA sequencing by in situ combinatorial indexing. Nat Microbiol. 2020;5(10):1192–1201. 10.1038/s41564-020-0729-6 PMC8330242 32451472

[67] Kuchina A, Brettner LM, Paleologu L, Roco CM, Rosenberg AB, Carignano A, Kibler R, Hirano M, Depaolo RW, Seelig G. Microbial single-cell RNA sequencing by split-pool barcoding. Science. 2021;371(6531): Article eaba5257. 10.1126/science.aba5257 PMC8269303 33335020

[68] Ma P, Amemiya HM, He LL, Gandhi SJ, Nicol R, Bhattacharyya RP, Smillie CS, Hung DT. Bacterial droplet-based single-cell RNA-seq reveals antibiotic-associated heterogeneous cellular states. Cell. 2023;186(4):877–891.e14. 10.1016/j.cell.2023.01.002 PMC10014032 36708705

[69] Wang B, Lin AE, Yuan J, Novak KE, Koch MD, Wingreen NS, Adamson B, Gitai Z. Single-cell massively-parallel multiplexed microbial sequencing (M3-seq) identifies rare bacterial populations and profiles phage infection. Nat Microbiol. 2023;8(10):1846–1862. 10.1038/s41564-023-01462-3 PMC10522482 37653008

[70] Blattman SB, Jiang W, Mcgarrigle ER, Liu M, Oikonomou P, Tavazoie S. Identification and genetic dissection of convergent persister cell states. Nature. 2024;636(8042):438–446. 10.1038/s41586-024-08124-2 PMC11634777 39506104

[71] Homberger C, Hayward RJ, Barquist L, Vogel J. Improved bacterial single-cell RNA-seq through automated MATQ-seq and Cas9-based removal of rRNA reads. MBio. 2023;14(2): Article e0355722. 10.1128/mbio.03557-22 PMC10127585 36880749

[72] Nishimura M, Takahashi K, Hosokawa M. Recent advances in single-cell RNA sequencing of bacteria: Techniques, challenges, and applications. J Biosci Bioeng. 2025;139(5):341–346. 10.1016/j.jbiosc.2025.01.008 39984340

[73] Mediati DG, Wu S, Wu W, Tree JJ. Networks of resistance: Small RNA control of antibiotic resistance. Trends Genet. 2021;37(1):35–45. 10.1016/j.tig.2020.08.016 32951948

[74] Cohen S, Rokach L, Veksler-Lublinsky I. GNNs and ensemble models enhance the prediction of new sRNA-mRNA interactions in unseen conditions. BMC Bioinformatics. 2025;26(1):131. 10.1186/s12859-025-06153-w PMC12093732 40399818

[75] Rajput A, Tsunemoto H, Sastry AV, Szubin R, Rychel K, Chauhan SM, Pogliano J, Palsson BO. Advanced transcriptomic analysis reveals the role of efflux pumps and media composition in antibiotic responses of Pseudomonas aeruginosa. Nucleic Acids Res. 2022;50(17):9675–9688. 10.1093/nar/gkac743 PMC9508857 36095122

[76] Gervasoni S, Mehla J, Bergen CR, Leus IV, Margiotta E, Malloci G, Bosin A, Vargiu AV, Lomovskaya O, Rybenkov VV, et al. Molecular determinants of avoidance and inhibition of Pseudomonas aeruginosa MexB efflux pump. MBio. 2023;14(4): Article e0140323. 10.1128/mbio.01403-23 PMC10470492 37493633

[77] Huang Y, Chen H, Wu J, Huang X, Zhang Y, Ren A, Wu X, Huang C, Xie W, Zu D, et al. Machine learning-driven discovery of aniline phenylacetate derivatives as LasR-targeted biofilm inhibitors against Pseudomonas aeruginosa infections. Acta Pharm Sin B. 2026. 10.1016/j.apsb.2026.03.048 PMC13712431 42948752

[78] Carneiro J, Magalhães RP, De La Oliva Roque VM, Simões M, Pratas D, Sousa SF. TargIDe: A machine-learning workflow for target identification of molecules with antibiofilm activity against Pseudomonas aeruginosa. J Comput Aided Mol Des. 2023;37(5):265–278. 10.1007/s10822-023-00505-5 PMC10232598 37085636

[79] Liu HY, Prentice EL, Webber MA. Mechanisms of antimicrobial resistance in biofilms. NPJ Antimicrob Resist. 2024;2(1):27. 10.1038/s44259-024-00046-3 PMC11445061 39364333

[80] Tjaden B. TargetRNA3: Predicting prokaryotic RNA regulatory targets with machine learning. Genome Biol. 2023;24(1):276. 10.1186/s13059-023-03117-2 PMC10691042 38041165

[81] Qian W, Sun J, Liu T, Yang Z, Tsui SK. sRNAdeep: A novel tool for bacterial sRNA prediction based on DistilBERT encoding mode and deep learning algorithms. BMC Genomics. 2024;25(1):1021. 10.1186/s12864-024-10951-6 PMC11526673 39482572

[82] Park HM, Park Y, Berani U, Bang E, Vankerschaver J, Van Messem A, De Neve W, Shim H. In silico optimization of RNA-protein interactions for CRISPR-Cas13-based antimicrobials. Biol Direct. 2022;17(1):27. 10.1186/s13062-022-00339-5 PMC9547417 36207756

[83] Zhu Y, Chen J, Shen H, Chen Z, Yang Q-W, Zhu J, Li X, Yang Q, Zhao F, Ji J, et al. Emergence of ceftazidime-and avibactam-resistant Klebsiella pneumoniae carbapenemase-producing Pseudomonas aeruginosa in China. Msystems. 2021;6(6): Article e0078721. 10.1128/mSystems.00787-21 PMC8562488 34726488

[84] Serafim MSM, Kronenberger T, Oliveira PR, Poso A, Honorio KM, Mota BEF, Maltarollo VG. The application of machine learning techniques to innovative antibacterial discovery and development. Expert Opin Drug Discov. 2020;15(10):1165–1180. 10.1080/17460441.2020.1776696 32552005

[85] Pun FW, Ozerov IV, Zhavoronkov A. AI-powered therapeutic target discovery. Trends Pharmacol Sci. 2023;44(9):561–572. 10.1016/j.tips.2023.06.010 37479540

[86] Özçelik R, Van Tilborg D, Jiménez-Luna J, Grisoni F. Structure-based drug discovery with deep learning. Chembiochem. 2023;24(13): Article e202200776. 10.1002/cbic.202200776 37014633

[87] Dhanda G, Acharya Y, Haldar J. Antibiotic adjuvants: A versatile approach to combat antibiotic resistance. ACS Omega. 2023;8(12):10757–10783. 10.1021/acsomega.3c00312 PMC10061514 37008128

[88] Mao J, Guan S, Chen Y, Zeb A, Sun Q, Lu R, Dong J, Wang J, Cao D. Application of a deep generative model produces novel and diverse functional peptides against microbial resistance. Comput Struct Biotechnol J. 2023;21:463–471. 10.1016/j.csbj.2022.12.029 PMC9804011 36618982

[89] Wang T, Zhang X, Zhang O, Chen G, Pan P, Wang E, Wang J, Wu J, Zhou D, Wang L, et al. Highly accurate and efficient deep learning paradigm for full-atom protein loop modeling with KarmaLoop. Research. 2024;7:0408. 10.34133/research.0408 PMC11268956 39055686

[90] Pang T, Zhang X, Zhou Y, Yang J. Integrated framework for targeting dynamic penicillin-binding protein 2a via ensemble structural biology and deep generative modeling. Front Microbiol. 2026;17:1811950. 10.3389/fmicb.2026.1811950 PMC13199089 42199546

[91] Sako M, Yasuo N, Sekijima M. Interaction-constrained 3D molecular generation using a diffusion model enables structure-based pharmacophore modeling for drug design. NPJ Drug Discov. 2026;3(1):8. 10.1038/s44386-026-00040-x PMC13267100 42380254

[92] Nyerges A, Tomašič T, Durcik M, Revesz T, Szili P, Draskovits G, Bogar F, Skok Ž, Zidar N, Ilaš J, et al. Rational design of balanced dual-targeting antibiotics with limited resistance. PLOS Biol. 2020;18(10): Article e3000819. 10.1371/journal.pbio.3000819 PMC7561186 33017402

[93] Fang Y, Jiang Y, Wei L, Ma Q, Ren Z, Yuan Q, Wei D-Q. DeepProSite: Structure-aware protein binding site prediction using ESMFold and pretrained language model. Bioinformatics. 2023;39(12): Article btad718. 10.1093/bioinformatics/btad718 PMC10723037 38015872

[94] Hart KM, Ho CM, Dutta S, Gross ML, Bowman GR. Modelling proteins’ hidden conformations to predict antibiotic resistance. Nat Commun. 2016;7:12965. 10.1038/ncomms12965 PMC5477488 27708258

[95] Srinivasan B, Rodrigues JV, Tonddast-Navaei S, Shakhnovich E, Skolnick J. Rational design of novel allosteric dihydrofolate reductase inhibitors showing antibacterial effects on drug-resistant Escherichia coli escape variants. ACS Chem Biol. 2017;12(7):1848–1857. 10.1021/acschembio.7b00175 PMC5819740 28525268

[96] Zhang Y, Chowdhury S, Rodrigues JV, Shakhnovich E. Development of antibacterial compounds that constrain evolutionary pathways to resistance. Elife. 2021;10: Article e64518. 10.7554/eLife.64518 PMC8331180 34279221

[97] Thomas CA, Alao JP, Smisek T, Cheng Z, Bethel CR, Yang K, Obaseki I, Page RC, Bonomo RA, Oelschlaeger P, et al. Inhibitor affinity differs among clinical variants of IMP metallo-β-lactamases: Analysis and implications for inhibitor design. ACS Infect Dis. 2025;11(8):2157–2168. 10.1021/acsinfecdis.5c00138 PMC12336985 40704846

[98] Ghosh S, Ramaiah S. A dual functional inhibitory molecule to combat GyrA-ParC and associated mutants in fluoroquinolone-resistant Shigella flexneri causing shigellosis: An ML guided and quantum simulation-based in silico approach. Front Bioinform. 2026;6:1765216. 10.3389/fbinf.2026.1765216 PMC13291547 42358624

[99] Tucs A, Tran DP, Yumoto A, Ito Y, Uzawa T, Tsuda K. Generating ampicillin-level antimicrobial peptides with activity-aware generative adversarial networks. ACS Omega. 2020;5(36):22847–22851. 10.1021/acsomega.0c02088 PMC7495458 32954133

[100] Lin T-T, Yang L-Y, Lin C-Y, Wang C-T, Lai C-W, Ko C-F, Shih Y-H, Chen S-H. Intelligent de novo design of novel antimicrobial peptides against antibiotic-resistant bacteria strains. Int J Mol Sci. 2023;24(7):6788. 10.3390/ijms24076788 PMC10095442 37047760

[101] Wang J, Feng J, Kang Y, Pan P, Ge J, Wang Y, Wang M, Wu Z, Zhang X, Yu J, et al. Discovery of antimicrobial peptides with notable antibacterial potency by an LLM-based foundation model. Sci Adv. 2025;11(10): Article eads8932. 10.1126/sciadv.ads8932 PMC13109931 40043127

[102] Yu Y, Zhang Z, Gu M, Yan W, Han J, Li R, Wei L, Ren X, Tian J, Xu S, et al. Rapid response antimicrobial peptide design strategy driven by meta-learning for emerging drug-resistant pathogens. J Med Chem. 2025;68(8):8530–8542. 10.1021/acs.jmedchem.5c00188 40193623

[103] Mendes B, Almeida JR, Castelletto V, Hamley IW, Barrett G. Harnessing snake venom cardiotoxins for antimicrobial peptide discovery. NPJ Drug Discov. 2026;3(1):40. 10.1038/s44386-026-00073-2 PMC13582888 42749748

[104] Schneuing A, Harris C, Du Y, Didi K, Jamasb A, Igashov I, Du W, Gomes C, Blundell TL, Lio P, et al. Structure-based drug design with equivariant diffusion models. Nat Comput Sci. 2024;4(12):899–909. 10.1038/s43588-024-00737-x PMC11659159 39653846

[105] Gao W, Coley CW. The synthesizability of molecules proposed by generative models. J Chem Inf Model. 2020;60(12):5714–5723. 10.1021/acs.jcim.0c00174 32250616

[106] Popova M, Isayev O, Tropsha A. Deep reinforcement learning for de novo drug design. Sci Adv. 2018;4(7): Article eaap7885. 10.1126/sciadv.aap7885 PMC6059760 30050984

[107] Ståhl N, Falkman G, Karlsson A, Mathiason G, Bostrom J. Deep reinforcement learning for multiparameter optimization in de novo drug design. J Chem Inf Model. 2019;59(7):3166–3176. 10.1021/acs.jcim.9b00325 31273995

[108] Dong R, Cao Q, Song C. Painting peptides with antimicrobial potency through deep reinforcement learning. Adv Sci. 2025;12(43): Article e06332. 10.1002/advs.202506332 PMC12631831 40940628

[109] Antonoplis A, Zang X, Huttner MA, Chong KK, Lee YB, Co JY, Amieva MR, Kline KA, Wender PA, Cegelski L. A dual-function antibiotic-transporter conjugate exhibits superior activity in sterilizing MRSA biofilms and killing persister cells. J Am Chem Soc. 2018;140(47):16140–16151. 10.1021/jacs.8b08711 PMC6430714 30388366

[110] Al-Wahaibi LH, Mahmoud MA, Alzahrani HA, Abou-Zied HA, Abdelmoez A, Youssif BGM, Bräse S, Rabea SM. Synthesis, enzyme inhibition, and docking studies of new schiff bases of disalicylic acid methylene-based derivatives as dual-target antibacterial agents. Front Chem. 2024;12:1493906. 10.3389/fchem.2024.1493906 PMC11590026 39600312

[111] Bell IP, Muniyan R. Targeting the quorum sensing network in Acinetobacter baumannii: A dual target structure-based approach for the development of novel antimicrobials. Comput Biol Med. 2025;187: Article 109828. 10.1016/j.compbiomed.2025.109828 39938338

[112] Xu J, Li F, Li C, Guo X, Landersdorfer C, Shen HH, Peleg AY, Li J, Imoto S, Yao J, et al. iAMPCN: A deep-learning approach for identifying antimicrobial peptides and their functional activities. Brief Bioinform. 2023;24(4): Article bbad240. 10.1093/bib/bbad240 PMC10359087 37369638

[113] Wu Y, Wang C, Shen X, Chen Y, Wang H, Xu B, Zhu Z, Chen Y, Dai W, Huang Y, et al. Iterative discovery of potent polymeric antibiotics via multi-stage and multi-task learning against antimicrobial resistance. Nat Commun. 2026;17(1):1878. 10.1038/s41467-026-68682-z PMC12923562 41565665

[114] Li X, Gong H, Wang Y, Zhao Y, Li L, Bao P, Kong Q, Fu J, Wan B, Zhang Y, et al. De novo multi-mechanism antimicrobial peptide design via multimodal deep learning. Adv Sci. 2026;13(28): Article e15835. 10.1002/advs.202515835 PMC13185819 41801219

[115] Wan F, Torres MDT, Peng J, De La Fuente-Nunez C. Deep-learning-enabled antibiotic discovery through molecular de-extinction. Nat Biomed Eng. 2024;8(7):854–871. 10.1038/s41551-024-01201-x PMC11310081 38862735

[116] Zhang Z, Yan J, Huang Y, Liu Q, Chen E, Wang M, Zitnik M. Structure-based drug design with geometric deep learning: A comprehensive survey. ACM Comput Surv. 2025;58(5):1–35.

[117] Imrie F, Bradley AR, Van Der Schaar M, Deane CM. Deep generative models for 3D linker design. J Chem Inf Model. 2020;60(4):1983–1995. 10.1021/acs.jcim.9b01120 PMC7189367 32195587

[118] Igashov I, Stärk H, Vignac C, Schneuing A, Satorras VG, Frossard P, Welling M, Bronstein M, Correia B. Equivariant 3D-conditional diffusion model for molecular linker design. Nat Mach Intell. 2024;6(4):417–427.

[119] Chung CH, Chang DC, Rhoads NM, Shay MR, Srinivasan K, Okezue MA, Brunaugh AD,Chandrasekaran S. Transfer learning predicts species-specific drug interactions in emerging pathogens. bioRxiv. 2024. https://doi: 10.1101/2024.06.04.597386

[120] Cantrell JM, Chung CH, Chandrasekaran S. Machine learning to design antimicrobial combination therapies: Promises and pitfalls. Drug Discov Today. 2022;27(6):1639–1651. 10.1016/j.drudis.2022.04.006 35398560

[121] Chandrasekaran S, Cokol-Cakmak M, Sahin N, Yilancioglu K, Kazan H, Collins JJ, Cokol M. Chemogenomics and orthology-based design of antibiotic combination therapies. Mol Syst Biol. 2016;12(5):872. 10.15252/msb.20156777 PMC5289223 27222539

[122] Arora HS, Lev K, Robida A, Velmurugan R, Chandrasekaran S. A metabolism-informed neural network identifies pathways influencing the potency and toxicity of antimicrobial combinations. NPJ Drug Discov. 2026;3(1):11. 10.1038/s44386-026-00042-9 PMC13043290 41940069

[123] Cacace E, Kim V, Varik V, Knopp M, Tietgen M, Brauer-Nikonow A, Inecik K, Mateus A, Milanese A, Mårli MT, et al. Systematic analysis of drug combinations against Gram-positive bacteria. Nat Microbiol. 2023;8(11):2196–2212. 10.1038/s41564-023-01486-9 PMC10627819 37770760

[124] Wicha SG, Märtson AG, Nielsen EI, Koch BC, Friberg LE, Alffenaar JW, Minichmayr IK, International Society of Anti-Infective Pharmacology (ISAP), The PK/PD study group of the European Society of Clinical Microbiology, Infectious Diseases (EPASG). From therapeutic drug monitoring to model-informed precision dosing for antibiotics. Clin Pharmacol Ther. 2021;109(4):928–941. 10.1002/cpt.2202 33565627

[125] Darwich AS, Polasek TM, Aronson JK, Ogungbenro K, Wright DF, Achour B, Reny J-L, Daali Y, Eiermann B, Cook J, et al. Model-Informed precision dosing: Background, requirements, validation, implementation, and forward trajectory of individualizing drug therapy. Annu Rev Pharmacol Toxicol. 2021;61(1):225–245. 10.1146/annurev-pharmtox-033020-113257 33035445

[126] Smith NM, Lenhard JR, Boissonneault KR, Landersdorfer CB, Bulitta JB, Holden PN, Forrest A, Nation RL, Li J, Tsuji BT. Using machine learning to optimize antibiotic combinations: Dosing strategies for meropenem and polymyxin B against carbapenem-resistant Acinetobacter baumannii. Clin Microbiol Infect. 2020;26(9):1207–1213. 10.1016/j.cmi.2020.02.004 PMC7587610 32061797

[127] Smith NM, Nguyen TD, Lodise TP, Chen L, Kaur JN, Klem JF, Boissonneault KR, Holden PN, Roach DR, Tsuji BT. Machine learning-led optimization of combination therapy: Confronting the public health threat of extensively drug resistant gram-negative bacteria. Clin Pharmacol Ther. 2024;115(4):896–905. 10.1002/cpt.3134 38062797

[128] Garcia E, Diep JK, Sharma R, Hanafin PO, Abboud CS, Kaye KS, Li J, Velkov T, Rao GG. Evaluation strategies for triple-drug combinations against carbapenemase-producing Klebsiella pneumoniae in an in vitro hollow-fiber infection model. Clin Pharmacol Ther. 2021;109(4):1074–1080. 10.1002/cpt.2197 PMC8048493 33548079

[129] Liu G, Catacutan DB, Rathod K, Swanson K, Jin W, Mohammed JC, Chiappino-Pepe A, Syed SA, Fragis M, Rachwalski K, et al. Deep learning-guided discovery of an antibiotic targeting Acinetobacter baumannii. Nat Chem Biol. 2023;19(11):1342–1350. 10.1038/s41589-023-01349-8 37231267

[130] Ali T, Basit A, Karim AM, Lee JH, Jeon JH, Rehman SU, Lee SH. Mutation-based antibiotic resistance mechanism in methicillin-resistant Staphylococcus aureus clinical isolates. Pharmaceuticals. 2021;14(5):420. 10.3390/ph14050420 PMC8147353 34062812

[131] Liu J, Chen Y, Tang J, Xing X, Lin J-S, Sun J, Xing X-H, Li J, Zhang CY. Deep learning-driven integrated pipeline for de novo design and synthesis of antimicrobial peptides. NPJ Drug Discov. 2026;3(1):15. 10.1038/s44386-026-00045-6 PMC13267055 42380318

[132] Nordmann P, Poirel L. Epidemiology and diagnostics of carbapenem resistance in Gram-negative bacteria. Clin Infect Dis. 2019;69(Suppl 7):S521–S528. 10.1093/cid/ciz824 PMC6853758 31724045

[133] Chiu LW, Ku YE, Chan FY, Lie WN, Chao HJ, Wang SY, Shen WC, Chen HY. Machine learning algorithms to predict colistin-induced nephrotoxicity from electronic health records in patients with multidrug-resistant Gram-negative infection. Int J Antimicrob Agents. 2024;64(1): Article 107175. 10.1016/j.ijantimicag.2024.107175 38642812

[134] Yang D, Li Y, Li C, Zhang Q, Huang J, Li X, Gao P. Biochemical-knowledge-driven machine learning pipeline for generating potent antimicrobial peptides. Brief Bioinform. 2026;27(2): Article bbag115. 10.1093/bib/bbag115 PMC12998437 41849223

[135] Hossain E, Yousefi N. Computational paradigms for antimicrobial resistance prediction: Integrating multi-omics, structural modeling, and foundation artificial intelligence systems. Brief Bioinform. 2026;27(3):–bbag219. 10.1093/bib/bbag219 PMC13158128 42108632

[136] Lu J, Chen J, Liu C, Zeng Y, Sun Q, Li J, Shen Z, Chen S, Zhang R. Identification of antibiotic resistance and virulence-encoding factors in Klebsiella pneumoniae by Raman spectroscopy and deep learning. Microb Biotechnol. 2022;15(4):1270–1280. 10.1111/1751-7915.13960 PMC8966003 34843635

[137] Liu B, Gao J, Liu XF, Rao G, Luo J, Han P, Hu W, Zhang Z, Zhao Q, Han L, et al. Direct prediction of carbapenem resistance in Pseudomonas aeruginosa by whole genome sequencing and metagenomic sequencing. J Clin Microbiol. 2023;61(11):e00617–e00623. 10.1128/jcm.00617-23 PMC10662344 37823665

[138] Xu Y, Liu D, Han P, Wang H, Wang S, Gao J, Chen F, Zhou X, Deng K, Luo J, et al. Rapid inference of antibiotic resistance and susceptibility for Klebsiella pneumoniae by clinical shotgun metagenomic sequencing. Int J Antimicrob Agents. 2024;64(2): Article 107252. 10.1016/j.ijantimicag.2024.107252 38908534

[139] Weis C, Cuénod A, Rieck B, Dubuis O, Graf S, Lang C, Oberle M, Brackmann M, Søgaard KK, Osthoff M, et al. Direct antimicrobial resistance prediction from clinical MALDI-TOF mass spectra using machine learning. Nat Med. 2022;28(1):164–174. 10.1038/s41591-021-01619-9 35013613

[140] Sturm A, Jóźwiak G, Verge MP, Munch L, Cathomen G, Vocat A, Luraschi-Eggemann A, Orlando C, Fromm K, Delarze E, et al. Accurate and rapid antibiotic susceptibility testing using a machine learning-assisted nanomotion technology platform. Nat Commun. 2024;15(1):2037. 10.1038/s41467-024-46213-y PMC10948838 38499536

[141] Rhodes NJ, Rohani R, Yarnold PR, Pawlowski AE, Malczynski M, Qi C, Sutton SH, Zembower TR, Wunderink RG. Machine learning to stratify methicillin-resistant Staphylococcus aureus risk among hospitalized patients with community-acquired pneumonia. Antimicrob Agents Chemother. 2023;67(1): Article e0102322. 10.1128/aac.01023-22 PMC9872682 36472425

[142] Goldschmidt E, Rannon E, Bernstein D, Wasserman A, Roimi M, Shrot A, Coster D, Shamir R. Predicting appropriateness of antibiotic treatment among ICU patients with hospital-acquired infection. NPJ Digit Med. 2025;8(1):87. 10.1038/s41746-024-01426-9 PMC11802796 39915601

[143] Yelin I, Snitser O, Novich G, Katz R, Tal O, Parizade M, Chodick G, Koren G, Shalev V, Kishony R. Personal clinical history predicts antibiotic resistance of urinary tract infections. Nat Med. 2019;25(7):1143–1152. 10.1038/s41591-019-0503-6 PMC6962525 31273328

[144] Howard A, Hughes DM, Green PL, Velluva A, Gerada A, Maskell S, Buchan IE, Hope W. Personalised antimicrobial susceptibility testing with clinical prediction modelling informs appropriate antibiotic use. Nat Commun. 2024;15(1):9924. 10.1038/s41467-024-54192-3 PMC11582675 39572574

[145] Hendriksen RS, Munk P, Njage P, Van Bunnik B, Mcnally L, Lukjancenko O, Röder T, Nieuwenhuijse D, Pedersen SK, Kjeldgaard J, et al. Global monitoring of antimicrobial resistance based on metagenomics analyses of urban sewage. Nat Commun. 2019;10(1):1124. 10.1038/s41467-019-08853-3 PMC6408512 30850636

[146] Weaver DT, King ES, Maltas J, Scott JG. Reinforcement learning informs optimal treatment strategies to limit antibiotic resistance. Proc Natl Acad Sci USA. 2024;121(16): Article e2303165121. 10.1073/pnas.2303165121 PMC11032439 38607932

[147] Vihta KD, Pritchard E, Pouwels KB, Hopkins S, Guy RL, Henderson K, Chudasama D, Hope R, Muller-Pebody B, Walker AS, et al. Predicting future hospital antimicrobial resistance prevalence using machine learning. Commun Med (Lond). 2024;4(1):197. 10.1038/s43856-024-00606-8 PMC11467333 39390045

[148] Ellabaan MMH, Munck C, Porse A, Imamovic L, Sommer MOA. Forecasting the dissemination of antibiotic resistance genes across bacterial genomes. Nat Commun. 2021;12(1):2435. 10.1038/s41467-021-22757-1 PMC8065159 33893312

[149] Zhou H, Beltrán JF, Brito IL. Functions predict horizontal gene transfer and the emergence of antibiotic resistance. Sci Adv. 2021;7(43): Article eabj5056. 10.1126/sciadv.abj5056 PMC8535800 34678056

[150] Guan J, Chen Z, Wang H, Li W, Ye F, Li S. Machine learning prediction of antibiotic resistance in Gram-negative pneumonia: A clinical-genomic integrated model. BMC Microbiol. 2026;26(1):477. 10.1186/s12866-026-05019-2 PMC13191928 41957589

[151] Anahtar MN, Yang JH, Kanjilal S. Applications of machine learning to the problem of antimicrobial resistance: An emerging model for translational research. J Clin Microbiol. 2021;59(7): Article e0126020. 10.1128/JCM.01260-20 PMC8218744 33536291

[152] Howard A, Reza N, Green PL, Yin M, Duffy E, Mwandumba HC, Gerada A, Hope W. Artificial intelligence and infectious diseases: Tackling antimicrobial resistance, from personalised care to antibiotic discovery. Lancet Infect Dis. 2026;26(3):e181–e192. 10.1016/S1473-3099(25)00313-5 40972630

[153] Penke TJ, Hammack AT, Mcmillan LJ, Baker E, Wilcock P, Healy N, Wall MK, Chavez N, Wright I, Tuson HH, et al. High-throughput methods leveraging robotics and computer vision for the development of therapeutic phage cocktails. Nat Commun. 2026;17(1):2192. 10.1038/s41467-026-68684-x PMC12960834 41617693

[154] Weiner I, Kahan-Hanum M, Buchstab N, Zelcbuch L, Navok S, Sherman I, Nicenboim J, Axelrod T, Berko-Ashur D, Olshina M, et al. Phage therapy with nebulized cocktail BX004-A for chronic Pseudomonas aeruginosa infections in cystic fibrosis: A randomized first-in-human trial. Nat Commun. 2025;16(1):5579. 10.1038/s41467-025-60598-4 PMC12216271 40593506

[155] Mcpherson JK, Hurdle JG, Baker ML, Hussain T, Kumar A, Garey KW. The microbiome-restorative potential of ibezapolstat for the treatment of Clostridioides difficile infection is predicted through variant PolC-type DNA polymerase III in Lachnospiraceae and Oscillospiraceae. Antimicrob Agents Chemother. 2025;69(4): Article e0167924. 10.1128/aac.01679-24 PMC11963600 39982073

[156] Keith M, Park De La Torriente A, Chalka A, Vallejo-Trujillo A, Mcateer SP, Paterson GK, Low AS, Gally DL. Predictive phage therapy for Escherichia coli urinary tract infections: Cocktail selection for therapy based on machine learning models. Proc Natl Acad Sci USA. 2024;121(12): Article e2313574121. 10.1073/pnas.2313574121 PMC10962980 38478693

[157] Duplessis CA, Biswas B. A review of topical phage therapy for chronically infected wounds and preparations for a randomized adaptive clinical trial evaluating topical phage therapy in chronically infected diabetic foot ulcers. Antibiotics. 2020;9(7):377. 10.3390/antibiotics9070377 PMC7400337 32635429

[158] Yang J, Eyre DW, Lu L, Clifton DA. Interpretable machine learning-based decision support for prediction of antibiotic resistance for complicated urinary tract infections. NPJ Antimicrob Resist. 2023;1(1):14. 10.1038/s44259-023-00015-2 PMC11057209 38686216

[159] Sanapalli BKR, Palit S, Deshpande A, Tokala R, Sigalapalli DK, Sanapalli V. Artificial intelligence and the discovery of antibiotics: Reinventing with opportunities, challenges, and clinical translation. Antibiotics. 2026;15(2):233. 10.3390/antibiotics15020233 PMC12937474 41750530

[160] Wilczok D, Zhavoronkov A. Progress, pitfalls, and impact of AI-driven clinical trials. Clin Pharmacol Ther. 2025;117(4):887–890. 10.1002/cpt.3542 PMC11924158 39722473

[161] Kim JI, Maguire F, Tsang KK, Gouliouris T, Peacock SJ, Mcallister TA, Mcarthur AG, Beiko RG. Machine learning for antimicrobial resistance prediction: Current practice, limitations, and clinical perspective. Clin Microbiol Rev. 2022;35(3): Article e0017921. 10.1128/cmr.00179-21 PMC9491192 35612324

[162] Ardila CM, González-Arroyave D, Tobón S. Machine learning for predicting antimicrobial resistance in critical and high-priority pathogens: A systematic review considering antimicrobial susceptibility tests in real-world healthcare settings. PLOS ONE. 2025;20(2): Article e0319460. 10.1371/journal.pone.0319460 PMC11856330 39999193

[163] Warrier SP, Narasimhan V, Meijer E, Gütlin Y, Nolte O, Veeraraghavan B, Egli A. Uncloaking the black-box: The need for explainable artificial intelligence in clinical microbiology and infectious diseases applications. Front Public Health. 2026;14:1776922. 10.3389/fpubh.2026.1776922 PMC13082983 42007334

[164] Abduljalil JM, Ataide SF, Kwan AH. Antibacterial drug discovery: Deep learning successes and challenges through the structural biology lens. Comput Struct Biotechnol J. 2026;35(1):0008. 10.34133/csbj.0008 PMC13123415 42057777

[165] Roger C. Understanding antimicrobial pharmacokinetics in critically ill patients to optimize antimicrobial therapy: A narrative review. J Intensive Med. 2024;4(3):287–298. 10.1016/j.jointm.2023.12.007 PMC11258509 39035618

[166] Vamathevan J, Clark D, Czodrowski P, Dunham I, Ferran E, Lee G, Li B, Madabhushi A, Shah P, Spitzer M, et al. Applications of machine learning in drug discovery and development. Nat Rev Drug Discov. 2019;18(6):463–477. 10.1038/s41573-019-0024-5 PMC6552674 30976107

[167] Zheng S, Gu Y, Gu Y, Zhao Y, Li L, Wang M, Jiang R, Yu X, Chen T, Li J. Machine learning–enabled virtual screening indicates the anti-tuberculosis activity of aldoxorubicin and Quarfloxin with verification by molecular docking, molecular dynamics simulations, and biological evaluations. Brief Bioinform. 2025;26(1): Article bbae696. 10.1093/bib/bbae696 PMC11684895 39737570

[168] U.S. Food and Drug Administration. *Considerations for the use of artificial intelligence to support regulatory decision-making for drug and biological products: Draft guidance for industry and other interested parties*. Silver Spring (MD): U.S. Food and Drug Administration; 2025. https://www.fda.gov/regulatory-information/search-fda-guidance-documents/considerations-use-artificial-intelligence-support-regulatory-decision-making-drug-and-biological

[169] U.S. Food and Drug Administration. *Guiding principles of good AI practice in drug development*. Silver Spring (MD): U.S. Food and Drug Administration; 2026. https://www.fda.gov/about-fda/artificial-intelligence-drug-development/guiding-principles-good-ai-practice-drug-development

[170] European Medicines Agency. *Reflection paper on the use of artificial intelligence (AI) in the medicinal product lifecycle*. Amsterdam (Netherlands): European Medicines Agency; 2024. https://www.ema.europa.eu/en/use-artificial-intelligence-ai-medicinal-product-lifecycle-scientific-guideline

[171] Mediouni M, Makarenkov V, Diallo AB. Towards an interpretable machine learning model for predicting antimicrobial resistance. J Glob Antimicrob Resist. 2025;45:47–51. 10.1016/j.jgar.2025.08.011 40865765

[172] Schneider P, Walters WP, Plowright AT, Sieroka N, Listgarten J, Goodnow RA Jr, Fisher J, Jansen JM, Duca JS, Rush TS, et al. Rethinking drug design in the artificial intelligence era. Nat Rev Drug Discov. 2020;19(5):353–364. 10.1038/s41573-019-0050-3 31801986

[173] Lin T-H, Chung H-Y, Jian M Jr, Chang C-K, Perng C-L, Chang F-Y, Chen Y-H, Shang H-S. Implementing an AI-enhanced clinical decision support system for Stenotrophomonas maltophilia: A survey-based randomized controlled trial of antibiotic precision and impact on survival. Implement Sci. 2025;20(1):47. 10.1186/s13012-025-01453-4 PMC12551136 41137088

[174] World Health Organization. *Global guidance framework for the responsible use of the life sciences: Mitigating biorisks and governing dual-use research*. Geneva (Switzerland): World Health Organization; 2022. https://www.who.int/publications/i/item/9789240056107

[175] Rieke N, Hancox J, Li W, Milletarì F, Roth HR, Albarqouni S, Bakas S, Galtier MN, Landman BA, Maier-Hein K, et al. The future of digital health with federated learning. NPJ Digit Med. 2020;3:119. 10.1038/s41746-020-00323-1 PMC7490367 33015372

[176] World Health Organization. *Ethics and governance of artificial intelligence for health: WHO guidance*. Geneva: World Health Organization; 2021. https://www.who.int/publications/i/item/9789240029200

[177] World Health Organization. *Antimicrobial resistance: Accelerating national and global responses: WHO strategic and operational priorities to address drug-resistant bacterial infections in the human health sector, 2025–2035. Report by the Director-General. A77/5*. Geneva (Switzerland): World Health Organization; 2024. https://apps.who.int/gb/ebwha/pdf_files/WHA77/A77_5-en.pdf

[178] Kasse GE, Cosh SM, Humphries J, Islam MS. Leveraging artificial intelligence for One Health: Opportunities and challenges in tackling antimicrobial resistance-scoping review. One Health Outlook. 2025;7(1):51. 10.1186/s42522-025-00170-8 PMC12532937 41102753

[179] Talianu A, Fraser-Krauss O, Bolton W, Ming D, Zhu N, Hernandez B, Gilchrist M, Holmes A, Georgiou P, Rawson TM. Artificial intelligence for improving decision-making in bacterial infection management: A narrative review. J Antimicrob Chemother. 2026;81(1): Article dkaf470. 10.1093/jac/dkaf470 PMC12802947 41424120

[180] Clinical and Laboratory Standards Institute. *Performance standards for antimicrobial susceptibility testing. 36th ed. CLSI supplement M100*. Wayne (PA): Clinical and Laboratory Standards Institute; 2026. https://clsi.org/shop/standards/m100/

[181] World Health Organization. *GLASS manual for antimicrobial resistance surveillance in common bacteria causing human infection*. Geneva (Switzerland): World Health Organization; 2023. https://www.who.int/publications/i/item/9789240076600

[182] Kaissis GA, Makowski MR, Rückert D, Braren RF. Secure, privacy-preserving and federated machine learning in medical imaging. Nat Mach Intell. 2020;2(6):305–311.

[183] Salam MA, Al-Amin MY, Pawar JS, Akhter N, Lucy IB. Conventional methods and future trends in antimicrobial susceptibility testing. Saudi J Biol Sci. 2023;30(3): Article 103582. 10.1016/j.sjbs.2023.103582 PMC9958398 36852413

[184] Tariq SS, Haleem MS, Uddin R. Data-driven design and screening of novel Klebsiella pneumoniae carbapenemase-2 β-lactamase inhibitors using a generative CLM. RSC Adv. 2026;16(31):28845–28856. 10.1039/d6ra02379g PMC13213580 42211383

[185] Shroff T, Aina K, Maass C, Cipriano M, Lambrecht J, Tacke F, Mosig A, Loskill P. Studying metabolism with multi-organ chips: New tools for disease modelling, pharmacokinetics and pharmacodynamics. Open Biol. 2022;12(3): Article 210333. 10.1098/rsob.210333 PMC8889168 35232251

[186] Mcdonald K, Rodriguez A, Muthukrishnan G. Humanized mouse models of bacterial infections. Antibiotics. 2024;13(7):640. 10.3390/antibiotics13070640 PMC11273811 39061322

[187] Giacobbe DR, Grossi AA, Bassetti M, De La Fuente-Nunez C. The future of antibiotics and artificial intelligence: some thoughts from discovery to bedside. Infect Dis Ther. 2026;15(2):461–475. 10.1007/s40121-025-01288-y PMC12855690 41442104

[188] Collins GS, Moons KGM, Dhiman P, Riley RD, Beam AL, Van Calster B, Ghassemi M, Liu X, Reitsma JB, Van Smeden M, et al. TRIPOD+AI statement: Updated guidance for reporting clinical prediction models that use regression or machine learning methods. BMJ. 2024;385: Article e078378. 10.1136/bmj-2023-078378 PMC11019967 38626948

[189] Liu X, Cruz Rivera S, Moher D, Calvert MJ, Denniston AK, SPIRIT-AI and CONSORT-AI Working Group. Reporting guidelines for clinical trial reports for interventions involving artificial intelligence: The CONSORT-AI extension. Nat Med. 2020;26(9):1364–1374. 10.1038/s41591-020-1034-x PMC7598943 32908283

[190] Kulkarni SG, Green AG, Mann BC, Malatesta S, Kulkarni-Goodwin S, Cesare N, Mulaudzi S, Rawoot N, Warren RM, et al. Convolutional neural networks quantify antibiotic resistance in Mycobacterium tuberculosis with diagnostic grade accuracy and predict treatment response. Nat Commun. 2026;17(1):5667. 10.1038/s41467-026-72225-x PMC13315309 42031767

[191] Hernàndez-Carnerero À, Sànchez-Marrè M, Mora-Jiménez I, Soguero-Ruiz C, Martínez-Agüero S, Álvarez-Rodríguez J. Dimensionality reduction and ensemble of LSTMs for antimicrobial resistance prediction. Artif Intell Med. 2023;138: Article 102508. 10.1016/j.artmed.2023.102508 36990585

[192] Pallikkavaliyaveetil N, Chandrasekaran S. Small data, big challenges: Machine- and deep-learning strategies for data-limited drug discovery. Adv Drug Deliv Rev. 2026;229: Article 115762. 10.1016/j.addr.2025.115762 PMC13094559 41421504

[193] Nguyen HA, Peleg AY, Wisniewski JA, Wang X, Wang Z, Blakeway LV, Badoordeen GZ, Theegala R, Doan NQ, Parker MH, et al. AMR-GNN: A multi-representation graph neural network framework to enable genomic antimicrobial resistance prediction. Nat Commun. 2026;17(1):3555. 10.1038/s41467-026-69934-8 PMC13087051 41792137

