# Supplementary material for: Artificial Intelligence as a Multifaceted Toolkit for Antibacterial Drug Discovery against Resistance
Source: Research (Wash D C). 2026 Nov 10;9:1457. doi: 10.34133/research.1457 (PMC13748630; doi:10.34133/research.1457)
Supplement: Supplementary file 1 [file research.1457.f1.docx]

**Supplemental Table 1.** Comparison of the present review with recent reviews on AI, antibacterial discovery, and antibacteriall resistance.

| Comparison Item | This Review | David *et al.* (2021) [1] | Liu *et al.* (2024) [2] | Howard *et al.* (2026) [3] | Melo et al. (2021) [4] | Sanapalli *et al.* (2026) [5] |
| --- | --- | --- | --- | --- | --- | --- |
| Review Type and Literature Search Scheme | Narrative review with a structured literature; Search Databases: PubMed, Web of Science, Scopus Search; Period: Jan 2018 - Apr 2025; Keywords: AI, machine learning, MDR bacteria, antibacterial agents; Inclusion: AI studies with bacterial *in vitro/in vivo* validation; Exclusion: Pure computational simulation without strain experiments | Narrative review; No unified retrieval rules, no fixed screening criteria | Narrative review; Partial databases mentioned, missing time and filter standards | Semi-systematic review; Only retrieved stewardship and diagnostic papers | Narrative review; No formal search, covers small molecules and AMPs | Narrative review; No formal search, broad AI antibiotic discovery |
| Core Classification Framework | Three-tier system: resistance mechanism analysis / molecular generation / efficacy prediction | Only lists small-molecule and AMP research cases | Mixed AI tools without systematic classification | Focuses on clinical CDSS and surveillance | Organized by ML pipeline: representation, prediction, design | By application: VS, PK, resistance prediction, AMP design |
| AMR Mechanism Coverage | Heteroresistance, persister cells, horizontal gene transfer and classic resistance pathways | Only conventional target mutation mechanisms | Partial Gram-negative resistance types covered | Rare discussion of molecular resistance | AMR context only; Target mutation mechanisms | Resistance prediction as a major topic |
| Distinctive Focus | 1. Two standardized comparison tables | Molecules and AMPs | AI across AMR | Clinical AI use | ML design and open science | Clinical focus |
|  | 2. Phased AI R&D deployment roadmap |  |  |  |  |  |
|  | 3. Comprehensive discussion of translation and regulation |  |  |  |  |  |
| Translational & Regulatory Discussion | Covers clinical trial standards, AI medical device regulations, data bias in low-resource regions | Rare content about preclinical and clinical transformation | Brief summary of preclinical results | Only hospital application discussion | Minimal translational content; no regulatory discussion | Addresses translational gaps; ethics and regulation discussed |

**References:**

1.	David L, Brata AM, Mogosan C, Pop C, Czako Z, Muresan L, Ismaiel A, Dumitrascu DI, Leucuta DC, Stanculete MF, et al. Artificial Intelligence and Antibiotic Discovery. *Antibiotics (Basel)*, 2021;10(11):1376

2.	Liu GY, Yu D, Fan MM, Zhang X, Jin ZY, Tang C,Liu XF. Antimicrobial resistance crisis: could artificial intelligence be the solution? *Mil Med Res*, 2024;11(1):7

3.	Howard A, Reza N, Green PL, Yin M, Duffy E, Mwandumba HC, Gerada A,Hope W. Artificial intelligence and infectious diseases: tackling antimicrobial resistance, from personalised care to antibiotic discovery. *Lancet Infect Dis*, 2026;26(3):e181-e192

4.	Melo MC, Maasch JR,De La Fuente-Nunez C. Accelerating antibiotic discovery through artificial intelligence. *Commun Biol*, 2021;4(1):1050

5.	Sanapalli BKR, Palit S, Deshpande A, Tokala R, Sigalapalli DK,Sanapalli V. Artificial Intelligence and the Discovery of Antibiotics: Reinventing with Opportunities, Challenges, and Clinical Translation. *Antibiotics (Basel)*, 2026;15(2):233
